# Blockade of the amino acid transporter SLC6A14 suppresses tumor growth in colorectal Cancer

**DOI:** 10.1186/s12885-022-09935-0

**Published:** 2022-07-30

**Authors:** Ying Lu, Ziting Jiang, Kaijing Wang, Shanshan Yu, Chongbo Hao, Zuan Ma, Xuelian Fu, Ming Qing Qin, Zengguang Xu, Lieying Fan

**Affiliations:** 1 Research Center for Translational Medicine, Shanghai East Hospital, School of Medicine, Tongji University, 150 Jimo Road, Pudong, Shanghai, 200120 China; 2 Shanghai East Hospital Ji’an Hospital, 80 Ji’an South Road, Ji’an City, 343000 Jiangxi Province China; 3 Department of Endoscopy, Fudan University Shanghai Cancer Center; Department of Oncology, Shanghai Medical College, Fudan University, Shanghai, 200032 China; 4 Department of Clinical Laboratory, Shanghai East Hospital, School of Medicine, Tongji University, 150 Jimo Road, Pudong, Shanghai, 200120 China

**Keywords:** SLC6A14, Colorectal cancer, Amino acid transporter, α-Methyltryptophan, mTOR

## Abstract

**Background:**

The amino acid transporter SLC6A14, which transports 18 of the 20 proteinogenic amino acids, is too low to be detected in healthy normal tissues but is significantly increased in some solid cancers. However, little is known about the roles of SLC6A14 in colorectal cancer (CRC).

**Methods:**

The mRNA and protein levels of SLC6A14 were detected using TCGA database, real-time polymerase chain reaction, western blot, and tissue microarrays, respectively. Amino acids concentration was determined by LC-MS/MS. Cell proliferation and apoptosis were determined using MTT assay and flow cytometry. Transwell invasion assay and wound healing assay were employed to analyze cell migration and invasion. The protein levels of Akt-mTOR signaling pathway and MMPs proteins were detected by western blot.

**Results:**

Both of the mRNA and protein levels of SLC6A14 were upregulated in CRC tissues, and the protein levels of SLC6A14 were closely related to the tumor cells differentiation: the higher the expression of SLC6A14 was, the poorer the differentiation of the tumor cells was. Further knockdown SLC6A14 with siRNA or treatment with α-MT in CRC cell lines reduced cell proliferation and migration in vitro and inhibited xenograft tumor growth in vivo. Mechanistically, SLC6A14 was demonstrated to regulate the expression and phosphorylation of Akt-mTOR, which mediates the promoting tumor growth function of SLC6A14. Blockade of SLC6A14 with α-MT inhibited the activation of mTOR signaling.

**Conclusion:**

SLC6A14 was upregulated in CRC and could promote tumor progression by activating the Akt-mTOR signaling pathway, which may serve as an effective molecular target for the treatment of CRC.

**Supplementary Information:**

The online version contains supplementary material available at 10.1186/s12885-022-09935-0.

## Background

Colorectal cancer (CRC) is the fourth cancer leading cause of death worldwide, and the morbidity and mortality rates have increased in recent years [1]. Up to now, the treatment of CRC is still based on early detection and surgical resection, while the treatment strategies for recurrence and metastasis of CRC patients are still limited. Moreover, targeted drugs are only effective in less than 20% of metastatic colorectal cancers [2, 3]. Therefore, it is important to find new targets for targeted therapy for CRC. Nowadays, gene microarrays, protein microarrays, and tumor metabonomics are all important means for identification of tumor markers, serodiagnosis, and new targeted therapies [4, 5].

Rapid proliferation of cancer cells increases the requirement for nutrients. Amino acids are essential for protein and nucleotide synthesis. However, mammalian cells cannot synthesize essential amino acids and need specific transporters to obtain them from the extracellular medium via the plasma membrane [6]. The expression of many different amino acid transporters is upregulated on the surface of tumor cells based on their molecular signature and metabolic profile to support increased biosynthesis [7, 8]. Recent interests have focused on amino acid transport and utilization in the field of tumor cell metabolism [9]: SLC1A5, alanine/serine/cysteine transporter 2, is upregulated in various cancers [10, 11]; SLC7A5, high affinity transporter of many neutral amino acids, is significantly upregulated in pancreatic cancer and hepatocellular carcinoma [12]; SLC7A11 mediates the influx of cystine, accompanied by the efflux of glutamate, and its upregulation protects cancer cells from oxidative stress [13, 14]. Moreover, the upregulation of SLC7A11 is often caused by deletion or inactivation of p53 [15]. Our previous studies have shown that the arginine transporter SLC7A1 is overexpressed in colorectal cancer tissues and is associated with cell growth [16].

SLC6A14/ATB^0,+^ transports all neutral amino acids and the two cationic amino acids, a total of 18 proteinogenic amino acids. Three different driving forces are involved in the activation of this transporter: a Na^+^ gradient, a Cl^−^ gradient, and a membrane potential. The stoichiometric ratio of Na^+^/Cl ^−^/amino acid is 2:1:1, because its transport function is coupled with Na^+^ and Cl^−^ [17]. Due to its broad substrate selectivity, SLC6A14 is an ideal transporter to meet the increased needs for amino acids in cancer cells [18]. SLC6A14 expression is too low to be detected in healthy tissues but is significantly increased in pancreatic, cervical, and breast cancers [19–21]. SLC6A14 knockout mice showed a low tumor incidence with normal fertility and mammary gland development. Moreover, many immunoglobulin genes were upregulated in SLC6A14^−/−^ mice, suggesting that the antitumor immune response might be stimulated [22]. Molecular characterization of SLC6A14 in transgenic models of breast cancer showed that CCAAT-enhancer binding protein homologous protein and asparagine synthetase were upregulated and might be responsible for the poor growth of tumors. α-Methyltryptophan (α-MT) has been identified as a pharmacologic blocker for SLC6A14 with minimal toxicity [23]. All these functions of SLC6A14 indicate that it deserves more attention as a new and effective method to target the anabolism of cancer cells. Although the upregulation of SLC6A14 was reported in CRC, because of the small sample size, the roles of SLC6A14 in CRC, the relationship between SLC6A14 and the clinicopathological characteristics of CRC and the underlying mechanisms were not fully understood [24, 25].

Therefore, we investigated the role of SLC6A14 in the pathogenesis of CRC and detected its clinicopathological correlation through four hundred and eighty-two tissue samples in this study. We found the effects of SLC6A14 on tumor cell growth in vitro and tumorigenesis in vivo, and confirmed that Akt-mTOR signaling was activated in SLC6A14-mediated CRC progression. Furthermore, we identified α-MT as an effective inhibitor targeting SLC6A14 to suppress tumor growth in CRC.

## Methods

### Patient samples and ethical approval

Four hundred and eighty-two tissue samples and twenty-eight pairs of human colorectal cancer and adjacent normal tissues were obtained from patients who underwent surgery at Shanghai East Hospital of Tongji University. All included patients met the following inclusion criteria: (1) pathological diagnoses were confirmed; (2) no preoperative chemotherapy or radiotherapy; (3) complete clinical data; and (4) informed consent was obtained. Exclusion criteria: (1) Patients with other primary tumors; (2) CRC recurrence patients with a second operation; (3) Follow-up data were missing. Normal tissues that were at least 5 cm away from the tumor were obtained from the patients. CRC specimens were staged in accordance with the 2010 American Joint Committee on Cancer staging system. The whole study was approved by the Ethics Committee of Shanghai East Hospital (No. 2018051).

### Cell lines and reagents

Human colorectal cancer cell lines (HT29, SW480, SW620, HCT116, and Caco2), and human liver cancer cell line Huh7 were provided by the Cell Bank of the Chinese Academy of Sciences (Shanghai, China). All cell lines were cultured in Dulbecco’s modified Eagle’s medium (DMEM) supplemented with 10% fetal calf serum (FBS) and incubated in a humidified chamber with 5% CO_2_ at 37 °C. The seeding densities of Caco2 cells were no more than 1 × 10^6^/mL. All experiments were performed using Caco2 cultured < 10 days post-seeding and the cell densities < 80%. DMEM and FBS were purchased from ThermoFisher Scientific, MA, USA. α-MT was purchased from Sigma-Aldrich, MO, USA.

### RNA extraction and real-time PCR

Tissue samples were lysed with TRIzol™ (ThermoFisher Scientific, MA, USA) and total RNA was extracted. Reverse transcription was performed using the first-strand cDNA Synthesis Kit (TaKaRa, Kyoto, Janpan) according to the manufacturer’s instructions. Real-time polymerase chain reaction (real-time PCR) was performed using SYBR Premix Ex Taq II PCR (TaKaRa, Kyoto, Japan) on ABI Q6 (Applied Biosystems, CA, USA) according to the manufacturer’s instructions. The sequences of the primers are shown in Supplementary Table 1.

### Tissue microarrays and immunohistochemical analysis

Four hundred and eighty-two tissue samples were obtained and used to generate tissue microarrays. The tissue sections were treated with immunohistochemical staining. Sections were heated in the Dako Target Retrieval Solution (Dako, Copenhagen, Denmark) at 95 °C for 20 minutes. After blocking with 3% H_2_O_2_, the sections were incubated with the primary antibody (SLC6A14, Abcam, ab254786, rabbit) at 4 °C overnight. They were rinsed with phosphate-buffered saline (PBS) three times for 15 minutes each time and then incubated with the secondary antibody (mouse anti-rabbit IgG-HRP, Santa Cruz, sc-2357) at room temperature for 30 minutes. Finally, the sections were visualized by using the diaminobenzidine (DAB) substrate kit according to the manufacturer’s instructions. The results were scanned on a Pannoraminc viewer (3D HISTECH, Pannoramic MIDI) with Quant Center software. The histochemistry score (H-score) was calculated as follows: H-score = (percentage of cells of weak intensity × 1) + (percentage of cells of moderate intensity × 2) + (percentage of cells of strong intensity × 3) [26].

### SLC6A14 knockdown in CRC cell lines

To knock down SLC6A14 expression in CRC cell lines, we used two siRNA targeted to knockdown SLC6A14 (si-SLC6A14–981: sense 5′-GCAACUCUGGAGGGUGCUUTT-3′, antisense 5′-AAGCACCCUCCAGAGUUGCTT-3′; si-SLC6A14–1702:sense 5′-GGUGGAGAGCUUGCUGGUUTT-3′, antisense 5′-AACCAGCAAGCUCUCCACCTT-3′), and a nonsilencing (NC)-siRNA (GenePharma, Shanghai, China) as a control in HCT116 and Caco2 cell lines. SLC6A14-siRNA or NC-siRNA was transfected into HCT116 and Caco2 cells using Lipofectamine 2000 Reagent (ThermoFisher Scientific, 11,668–019) according to the manufacturer’s instructions. Forty-eight hours after transfection, HCT116 and Caco2 cells were used to determine their function.

### Tumorigenicity in vivo

BALB/c nude mice were divided into two groups: the control group was given sucrose-water (5%), and the experimental group was given 2 mg/ml of α-MT in sucrose-water for free drinking starting from 7 days prior to cancer cell injection [20, 21, 27]. In consistence with literature [20], DL-form of α-MT was used. Both groups of mice were subcutaneously injected with HCT116 cells (1 × 10^6^/mouse) in 100 μl of physiological saline (0.9% NaCl). In the whole process of the experiment, mice in the treatment group were given 2 mg/ml α-MT in sucrose-water continuously and the control group were given sucrose-water only. Tumor volumes were measured and calculated according to the following formula: (length x width^2^)/2. The width and length of the tumors in each group were measured with vernier caliper once every 3 days. At the end of each experiment, the animals were sacrificed by cervical dislocation and the tumors were isolated and weighted.

All experimental procedures were conducted in accordance with the Public Health Service Policy and complied with the ARRIVE guidelines for the humane use and care of animals. The Institutional Animal Care and Use Committee in Shanghai East Hospital of Tongji University approved the animal experiments reported in the present study (No.2019085).

### Western blot analysis

HCT116 or Caco2 cells transfected with siRNA or treated with 2.5 mM α-MT for 48 h were lysed by using RIPA buffer, and the total protein concentration was quantified by BCA assays (Beyotime, Inc., Shanghai, China). The lysates were separated by electrophoresis on a 10% or 8% SDS-PAGE gel and transferred onto a polyvinylidene fluoride (PVDF) membrane. The membranes were blocked with 5% milk (w/v) at room temperature for 1 h, cut into appropriate pieces according to molecular weight or full membranes and then incubated with specific primary antibodies at 4 °C overnight with gentle shaking. We used β-actin (1:1000, Santa Cruz, sc-8432, rabbit) as the loading control. After washed with PBST buffer three times, the membranes were then incubated with secondary antibodies (1:2000, mouse anti-rabbit IgG, Santa Cruz, sc-516,253) at room temperature for another 1 h. The bands of the proteins were visualized using the Odyssey Infrared Imaging System (Li-COR, USA). ImageJ software (National Institutes of Health, Bethesda, MD, USA) was used to perform all densitometric analyses of the bands. The antibodies used in this study included SLC6A14 (1:2000, Abcam, ab254786, rabbit), mTOR (1:1000, CST, 2983S, rabbit), phospho-mTOR (Ser2448) (1:1000, CST, 5536 T, rabbit), GβL (1:1000, CST, 3274 T, rabbit), raptor (1:1000, CST, 2114 T, rabbit), MMP-2 (1:1000, CST, 87809, rabbit), MMP-9 (1:1000, CST, 13667, rabbit), MT1-MMP (1:1000, CST, 13130S, rabbit), Phospho-p70s6 Kinase (1:1000, CST, 9204S, rabbit), Phospho-S6 Ribosomal Protein (1:1000, CST, 4858 T, rabbit), Phospho-4E-BP1 (1:1000, CST, 9456S, rabbit), Akt (1:1000, CST, 4685S, rabbit) and Phospho-Akt (Ser473) (1:1000, CST, 4060S, rabbit).

### Detection of amino acids by LC-MS/MS

Amino acids concentration in the culture supernatant of HCT116 cells was determined by LC-MS/MS. Briefly, HCT116 cells transfected with siRNA or pharmacological blockade with 2.5 mM α-MT were plated at 5 × 10^6^ cells/well in 6-well plates and cultured for 24 h. Collect the supernatant and inject it on an Agilent HILIC Plus RRHD column (Agilent, CA, USA) with 5 μl. The flow rate was held constant at 400 μl/min and amino acids were detected.

### Cell proliferation assays

MTT assay was performed to determine cell proliferation. Briefly, HCT116 or Caco2 cells transfected with siRNA and cultured in DMEM containing 2.5 mM α-MT were plated at 1 × 10^3^ cells/well in 96-well plates. At the end of each period, the CellTiter 96 Aqueous One Solution Cell Proliferation Assay Kit (Promega, WI, USA) was used according to the manufacturer’s instructions and incubated with HCT116 or Caco2 cells for 4 h at 37 °C. Then the optical density (OD) value was read at the wavelength of 570 nm on an automated plate reader.

### Flow cytometry analysis of the cell cycle and apoptosis

Flow cytometry was used to analyze the cell cycle and apoptosis. After collecting HCT116 or Caco2 cells transfected with siRNA or treated with 2.5 mM α-MT, a total of 1 × 10^6^ cells were fixed with 70% ice-cold ethanol for 24 h at 4 °C for cell cycle analysis. The cells were stained with 50 μg/ml propidium iodide containing 0.1 mg/ml RNase A at 37 °C for 15 minutes. The DNA content of the HCT116 or Caco2 cells was determined by BD FACSCalibur cytometry and analyzed by ModFit LT software (Verity Software House, ME, USA). HCT116 or Caco2 cells transfected with siRNA or treated with 2.5 mM α-MT were cultured in FBS-free media for 24 h to induce apoptosis. For the apoptosis analysis, HCT116 or Caco2 cells were collected and stained with Annexin V-FITC and PI Apoptosis Detection Kit (BD Bioscience, NJ, USA).

### Cell invasion assay

Cell invasion was measured by using Corning® BioCoat™ Matrigel® Invasion assay kit (Corning, NY, USA) according to the manufacturer’s instructions. HCT116 or Caco2 cells with 4×10^5^ cells/well transfected with siRNA or treated with 2.5 mM α-MT were seeded into the upper chambers in FBS-free media and incubated for 24 h, accompanied by DMEM containing 10% FBS was added to the bottom chambers. Invaded cells on the bottom surface were fixed, stained with 0.5% crystal violet, and counted in five randomly chosen visual fields.

### Wound healing assay

When HCT116 or Caco2 cells transfected with siRNA or treated with 2.5 mM α-MT were cultured to about 80% confluence in a 6-well plate, a wound about 800 μm width was created by scratching cells with a sterile 10 μl plastic tip. Cells were cultured with DMEM containing 0.5% FBS for 48 h and photographed.

### Statistical analysis

The quantitative data were shown as mean ± SD. Statistical differences between two groups were analyzed by two-tailed Student’s t-tests, and comparisons among three or more groups by one-way ANOVA, performed with the statistical software SPSS 17.0 (IBM Corporation, NY, USA). Only a *p* value of less than 0.05 was considered statistically significant.

## Results

### Identification of SLC6A14 as a potential target in CRC

**Fig. 1:**
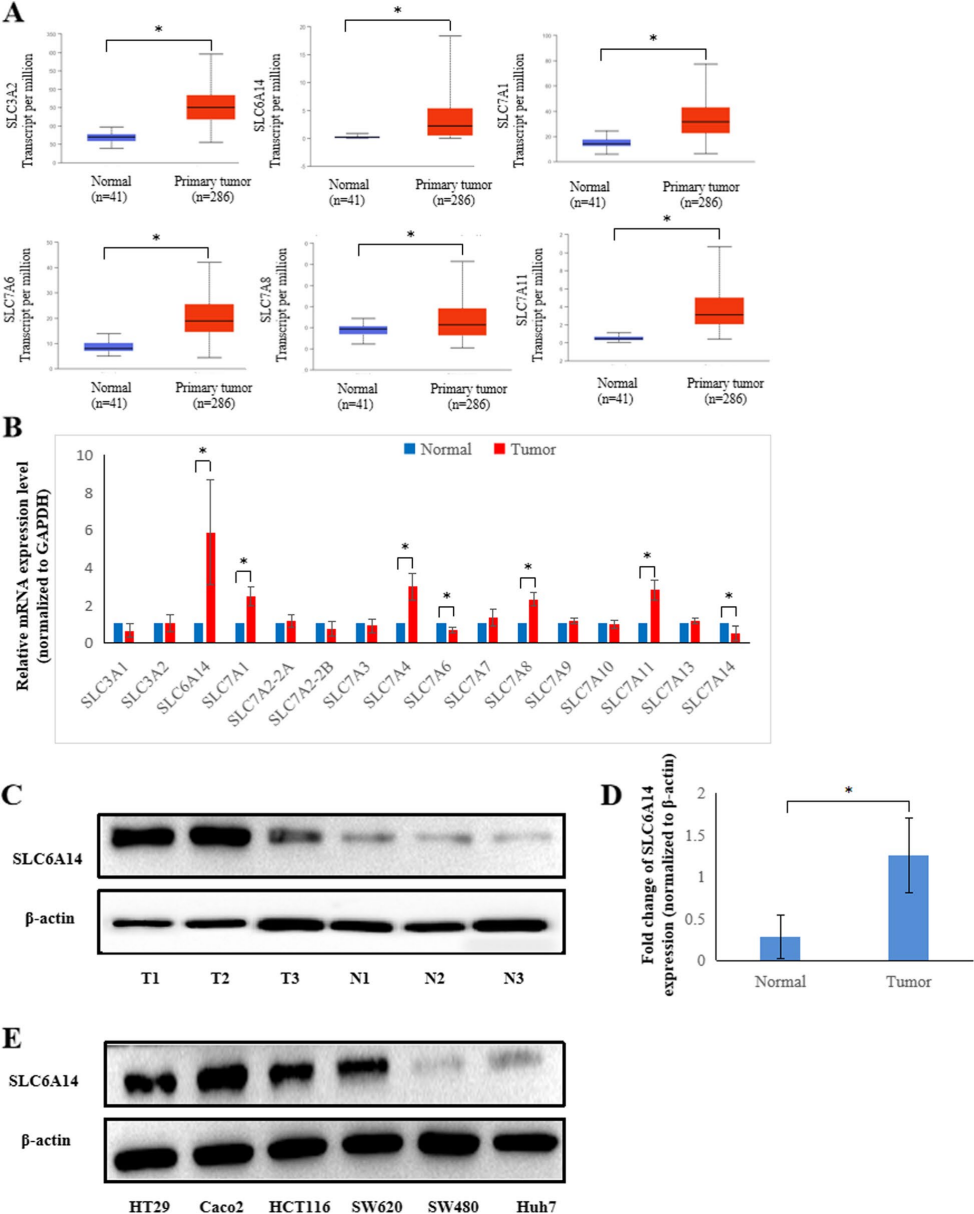
Amino acid transporter expression analysis showed that SLC6A14 is upregulated in colorectal cancer. **A** The publicly available TCGA database was used to analyze the mRNA expression levels of SLC6A14, SLC3, and SLC7 family transporters in colorectal cancer. **B** mRNA expression of SLC6A14, SLC3, and SLC7 family transporters in 28 matched CRC cancer tissues and paired adjacent colon tissues was detected by RT-PCR, and GAPDH was used as the internal control. The *P* value was calculated by Student’s test. **C** Representative images of SLC6A14 protein expression in paired normal and colorectal cancer tissues by Western blots. **D** The relative protein levels of SLC6A14 in the paired normal and colorectal cancer tissues were analyzed, and β-actin was used as the internal control. **E** The expression of SLC6A14 was evaluated by Western blots in five CRC cell lines (HT29, Caco2, HCT116, SW620 and SW480), and the liver cancer cell line Huh7. The data are expressed as the mean ± SD of three independent experiments. *P < 0.05

To determine the amino acid transporter expression profile in colon adenocarcinoma (COAD), we used the publicly available web tool UALCAN (website: http://ualcan.path.uab.edu/index.html) [28] with The Cancer Genome Atlas (TCGA) database to analyze the expression of common amino acid transporters (including SLC6A14, SLC3 and SLC7 families) in CRC and normal colonic tissue samples. Among the multiple amino acid transporters that exist in COAD, SLC3A2, SLC6A14, SLC7A1, SLC7A6, SLC7A8 and SLC7A11 have been shown to be upregulated (Fig. 1A). Nevertheless, SLC3A1, SLC7A2, SLC7A3, SLC7A4, and SLC7A14 were downregulated (**Supplementary Fig.** **1**). There was no significant difference in SLC7A7, SLC7A9, SLC7A10, and SLC7A13 in COAD (data not shown).

Next, we examined SLC6A14, SLC3 and SLC7 families mRNA expression in 28 paired CRC patient tissues by RT-PCR. The results showed that SLC6A14 expression was significantly higher in the cancer tissues than in the paired adjacent normal colon tissues (Fig. 1B). Consistent with the RT-PCR results, the protein expression of SLC6A14 in the CRC tissues was higher than that in the paired adjacent normal colon tissues, as shown in Fig. 1C and D. Furthermore, we found that SLC6A14 expression was substantially increased in the CRC cell lines (HT29, HCT116, SW620 and Caco2) compared to the liver cancer cell line (Huh7) (Fig. 1E).

### SLC6A14 was frequently upregulated in CRC and predicted poor differentiation

**Fig. 2:**
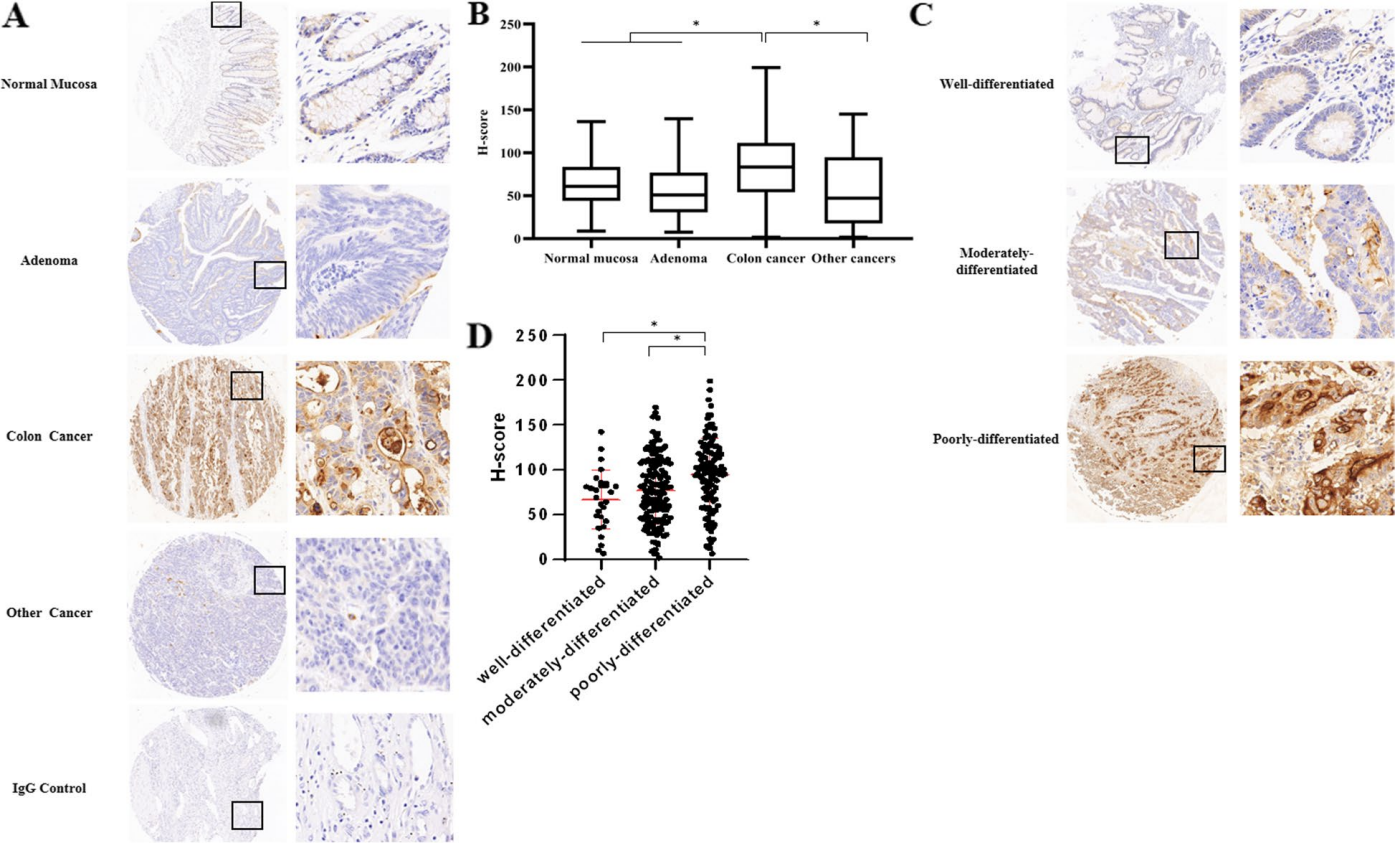
SLC6A14 was frequently upregulated in CRC and predicted poor differentiation. **A** Representative images of the protein expression of SLC6A14 generated from the immunohistochemistry-based tissue microarray containing 482 samples. **B** The protein expression of SLC6A14 was analyzed by H-score in 482 tissue samples: 94 normal mucosa, 65 adenoma, 302 colon cancer, and 21 liver cancer samples. **C** Representative images of the protein expression of SLC6A14 in different differentiation of CRC. **D** The H-score of SLC6A14 staining in well-differentiated, moderately differentiated, and poorly differentiated CRC. Comparisons among three or more groups were conducted using one-way ANOVA, *P < 0.05

To further explore the clinicopathological role of SLC6A14 in CRC progression, we assessed the protein expression of SLC6A14 in CRC tissue microarrays using immunohistochemistry staining. In the 482 cases, 302 CRC tissues showed higher SLC6A14 expression compared with the tissues from normal mucosa, adenoma, and liver cancer (Fig. 2A and B). Furthermore, as shown in Table 1**,** SLC6A14 expression was closely correlated with tumor differentiation: its expression in poorly differentiated tumor tissues was significantly higher than that in moderately and well-differentiated cancer tissues (*P* < 0.001, Fig. 2C and D). We also observed the positive correlation between SLC6A14 expression and clinical staging: the expression of SLC6A14 in stages III and IV was significantly higher than that in stages I and II (*P* < 0.001). However, there was no significant correlation among SLC6A14 expression and age, gender, tumor size or tumor location in our analysis.

**Table 1:** Relationship between the protein expression of SLC6A14 and clinicopathologic characteristics of colon cancer

Group	Number of cases	H scores	***t*** or ***F*** value	***P*** value
Pathological type				
Normal mucosa	94	63.26 ± 2.82	16.516	<0.001**
Adenoma	65	54.96 ± 3.77		
Colon cancer	302	83.15 ± 2.26		
Other types (liver cancer)	21	57.14 ± 9.78		
Age (year-old)				
≤ 60	90	82.07 ± 3.85		
> 60	212	83.93 ± 2.75	0.378	0.706
Gender				
Male	168	86.33 ± 3.05		
Female	134	79.67 ± 3.29	1.476	0.141
Tumor size (cm)				
≤ 5	192	80.76 ± 2.75		
> 5	110	87.94 ± 3.85	1.544	0.124
Tumor location				
Colon	182	81.33 ± 2.93		
Rectum	120	86.47 ± 3.51	1.120	0.264
Differentiation				
Well-differentiated	28	67.05 ± 6.24		
Moderately-differentiated	153	76.25 ± 3.00		
Poorly-differentiated	121	95.60 ± 3.64	11.556	<0.001**
Clinical stage				
I + II	84	69.79 ± 4.37		
III + IV	218	97.81 ± 2.92	−4.962	<0.001**

**P <0.001

### SLC6A14 promotes CRC cell growth and inhibits apoptosis by transporting amino acids

**Fig. 3:**
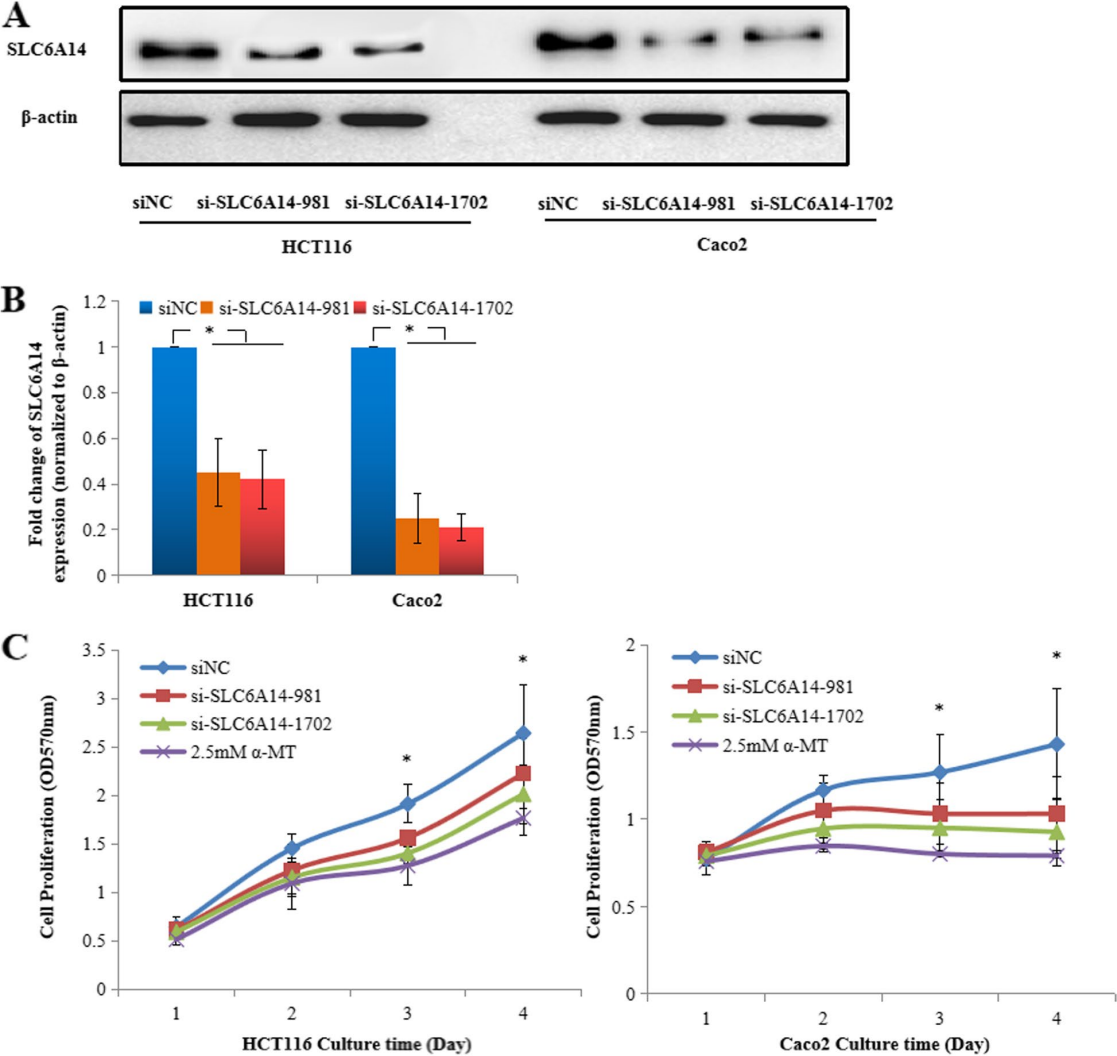
Knockdown or blockade of SLC6A14 inhibits cell growth. **A** Decreased protein expression of SLC6A14 in the HCT116 and Caco2 cells treated with siRNA was detected by western blots. **B** The relative protein levels of SLC6A14 in the HCT116 and Caco2 cells treated with siRNA were analyzed, and β-actin was used as the internal control. **C** The growth curve of the HCT116 and Caco2 cells that were transfected with SLC6A14-siRNA or blockade of SLC6A14 with α-MT, as determined by MTT assay. The data are expressed as the mean ± SD of three independent experiments. *P < 0.05

To determine the biological function of SLC6A14 in CRC, we performed cell proliferation assays, cell apoptosis assays, and cell cycle analysis of HCT116 and Caco2 cells with high SLC6A14 expression. α-Methyltryptophan (α-MT) is an inhibitor of SLC6A14. Western blot analysis showed that the protein expression of SLC6A14 in HCT116 or Caco2 cells after knocking down of SLC6A14 with siRNA was significantly decreased (Fig. 3A and B). LC-MS/MS was used to determine 20 amino acids concentration in the culture supernatant of HCT116 cells and found that 16 amino acids (except L-histidine, L-ornithine, L-citrulline, and L-aspartic acid) concentrations were increased in the culture supernatant of HCT116 cells by silencing SLC6A14 with siRNA or pharmacological blockade of SLC6A14 with α-MT, indicating that SLC6A14 plays an important role in amino acid uptake in the SLC6A14-positive cancer cells **(**Table 2**)**.

**Table 2:** The concentration of amino acids in the culture supernatant of HCT 116 cells treated with SLC6A14-siRNA or pharmacological blockade with α-MT for 24 h by LC-MS/MS (μmol/L)

Amino acid	siNC	si-SLC6A14–981	si-SLC6A14–1702	α-MT	***F*** value	***P*** value
L-Arg	980.0 ± 50.0	1100.0 ± 70.0	1230.0 ± 80.0	1250.0 ± 60.0	10.900	0.003*
L-Lys	95.4 ± 10.2	145.0 ± 11.3	167.0 ± 11.3	168.0 ± 14.2	24.720	<0.001**
L-His	79.3 ± 8.9	78.7 ± 8.7	78.4 ± 9.2	80.2 ± 12.3	0.019	0.996
L-Orn	29.6 ± 3.5	39.8 ± 4.7	37.9 ± 4.2	37.0 ± 5.5	2.918	0.100
L-Ala	147.0 ± 13.0	183.0 ± 15.0	223.0 ± 16.0	230.0 ± 21.0	16.330	<0.001**
L-Pro	162.0 ± 13.0	187.0 ± 15.0	208.0 ± 17.0	232.0 ± 24.0	8.485	0.007*
L-Thr	86.1 ± 8.5	132.0 ± 11.2	127.0 ± 12.2	129.0 ± 9.4	13.010	0.002*
L-Cit	5.4 ± 1.2	5.5 ± 1.3	6.1 ± 1.4	5.9 ± 2.6	0.125	0.943
Gly	182.0 ± 35.0	332.0 ± 46.0	270.0 ± 48.0	338.0 ± 34.0	9.276	0.006*
L-Ser	2.7 ± 1.5	17.4 ± 3.4	35.7 ± 4.2	23.4 ± 3.5	51.620	<0.001**
L-Asn	159.0 ± 23.0	309.0 ± 35.0	326.0 ± 32.0	336.0 ± 34.0	24.700	<0.001**
L-Tyr	57.9 ± 5.6	86.9 ± 8.7	102.0 ± 9.2	97.4 ± 10.3	15.800	0.001*
L-Leu	117.0 ± 23.0	203.0 ± 34.0	252.0 ± 25.0	268.0 ± 16.0	21.550	<0.001**
L-Glu	353.0 ± 28.0	432.0 ± 36.0	497.0 ± 39.0	475.0 ± 42.0	9.025	0.006*
L-Ile	99.3 ± 10.0	170.0 ± 12.0	185.0 ± 14.0	183.0 ± 14.0	31.050	<0.001**
L-Met	43.9 ± 5.4	57.7 ± 4.6	70.2 ± 6.3	78.4 ± 8.5	16.790	<0.001**
L-Trp	3.0 ± 1.2	6.3 ± 1.6	9.5 ± 2.4	9.5 ± 3.2	5.826	0.021*
L-Phe	47.3 ± 6.8	61.1 ± 8.2	95.9 ± 10.0	83.2 ± 8.2	20.310	<0.001**
L-Asp	106.0 ± 25.0	148.0 ± 24.0	168.0 ± 34.0	178.0 ± 38.0	3.591	0.066
L-Val	65.3 ± 8.2	121.0 ± 11.0	139.0 ± 12.0	142.0 ± 15.0	27.260	<0.001**

*P <0.05；**P <0.001

**Fig. 4:**
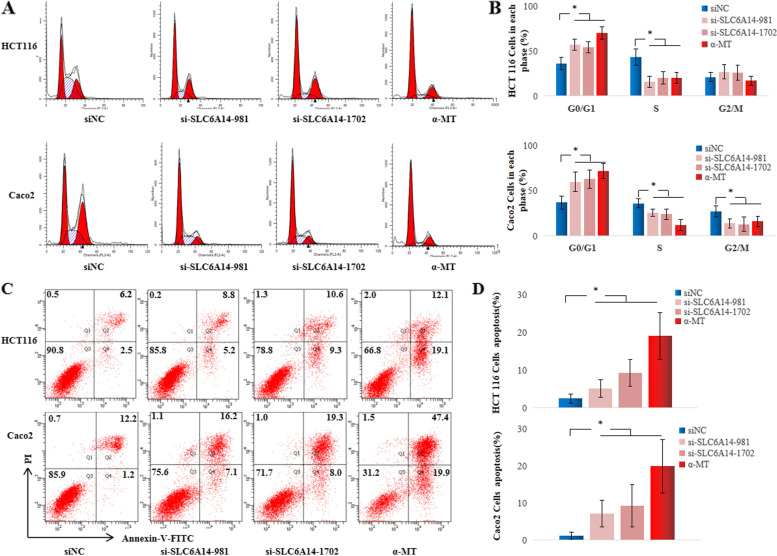
Knockdown or blockade of SLC6A14 suppresses cell proliferation and promotes apoptosis. **A** Cell cycle analysis G_1_/S transition of the HCT116 or Caco2 cells transfected with SLC6A14-siRNA or treated with α-MT by flow cytometry. **B** Quantification of cell cycle analysis. **C** The cell apoptosis of the HCT116 or Caco2 cells transfected with SLC6A14-siRNA or treated with α-MT cultured in FBS-free medium was stained with Annexin-V/PI and examined by flow cytometry. **D** The quantification of apoptotic cells identified as PI-negative and Annexin-V-positive staining. The data are expressed as the mean ± SD of three independent experiments. *P < 0.05

The MTT assay was used to assess cell growth after knockdown SLC6A14 or pharmacological blockade of SLC6A14 with α-MT. As shown in Fig. 3C, the HCT116 or Caco2 cells transfected with SLC6A14-siRNA or blockade with α-MT displayed significant growth inhibition compared with those transfected with NC-siRNA. We also measured cell cycle progression by flow cytometry analysis and found that HCT116 or Caco2 cells transfected with SLC6A14-siRNA or treated with α-MT showed a significant increase in G0/G1 phase and a significant decrease in the S phase compared with those transfected with NC-siRNA (Fig. 4A and B). Whether SLC6A14 affects cell apoptosis was also performed in FBS-free medium or medium with 10% FBS by flow cytometry analysis. Interestingly, the percent of early apoptotic cells significantly increased in the SLC6A14-siRNA-treated or blockade with α-MT cells compared with the NC-siRNA-treated cells (Fig. 4C and D**; Supplementary Fig.** **3**). However, the SW480 cells with low SLC6A14 expression treated with either SLC6A14-siRNA or α-MT blockade did not show any significant change of cell proliferation and cell apoptosis, compared with controls as shown in **Supplementary Fig.** **2**. These findings indicated that SLC6A14 promoted cell proliferation and viability in SLC6A14-positive CRC.

### SLC6A14 enhances the migration of CRC cells in vitro

**Fig. 5:**
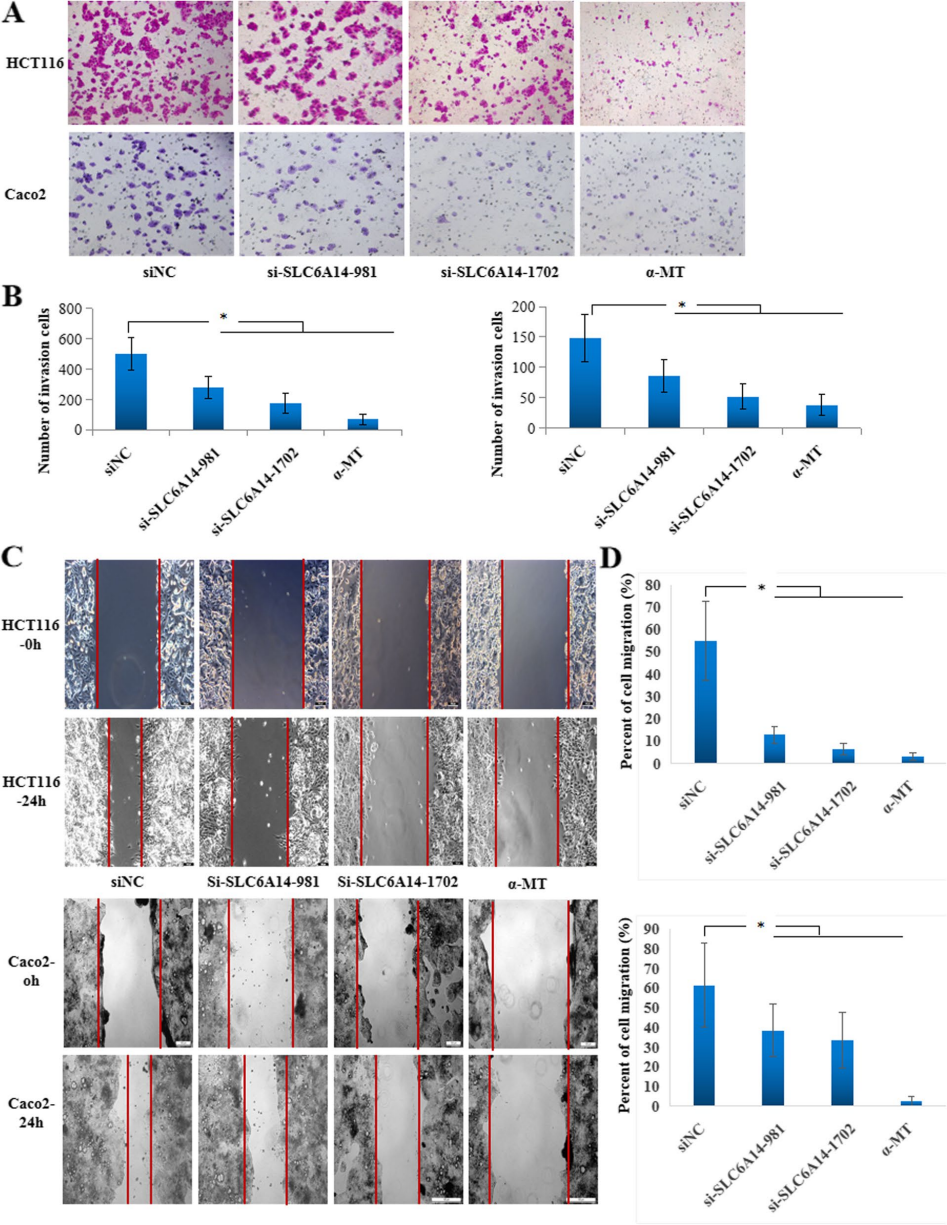
Knockdown or blockade of SLC6A14 reduced CRC cell motility. **A** The migratory abilities of the HCT116 or Caco2 cells transfected with SLC6A14-siRNA or blockade of SLC6A14 with α-MT were evaluated by transwell chamber assays. Representative fields of migrated cells are shown. **B** Quantification of cells. **C** A wound healing assay was used to analyze the motility of the HCT116 or Caco2 cells transfected with SLC6A14-siRNA or blockade of SLC6A14 with α-MT. The data are expressed as the mean ± SD of three independent experiments. **D** The percent of cell migration was calculated by Image J and the data are shown.*P < 0.05

To determine whether SLC6A14 plays a role in cell migration, we performed transwell assays and wound healing assays of HCT116 and Caco2 cells. Transwell assays data demonstrated that SLC6A14 knockdown significantly reduced cell invasion in both the HCT116 and Caco2 cells compared with the NC-siRNA-treated cells (Fig. 5A and B). Consistently, pharmacological blockade of SLC6A14 with α-MT achieved similar effects (Fig. 5A and B). Furthermore, a wound healing assay was employed to detect the effect of SLC6A14 on cell migration. As shown in Fig. 5C and D**,** knockdown or blockade of SLC6A14 restrained cell motility in the HCT116 and Caco2 cells. These results indicated that SLC6A14 plays an important role in CRC cell migration in vitro.

### SLC6A14 regulates the activities of the Akt-mTOR signaling pathway and MMPs

**Fig. 6:**
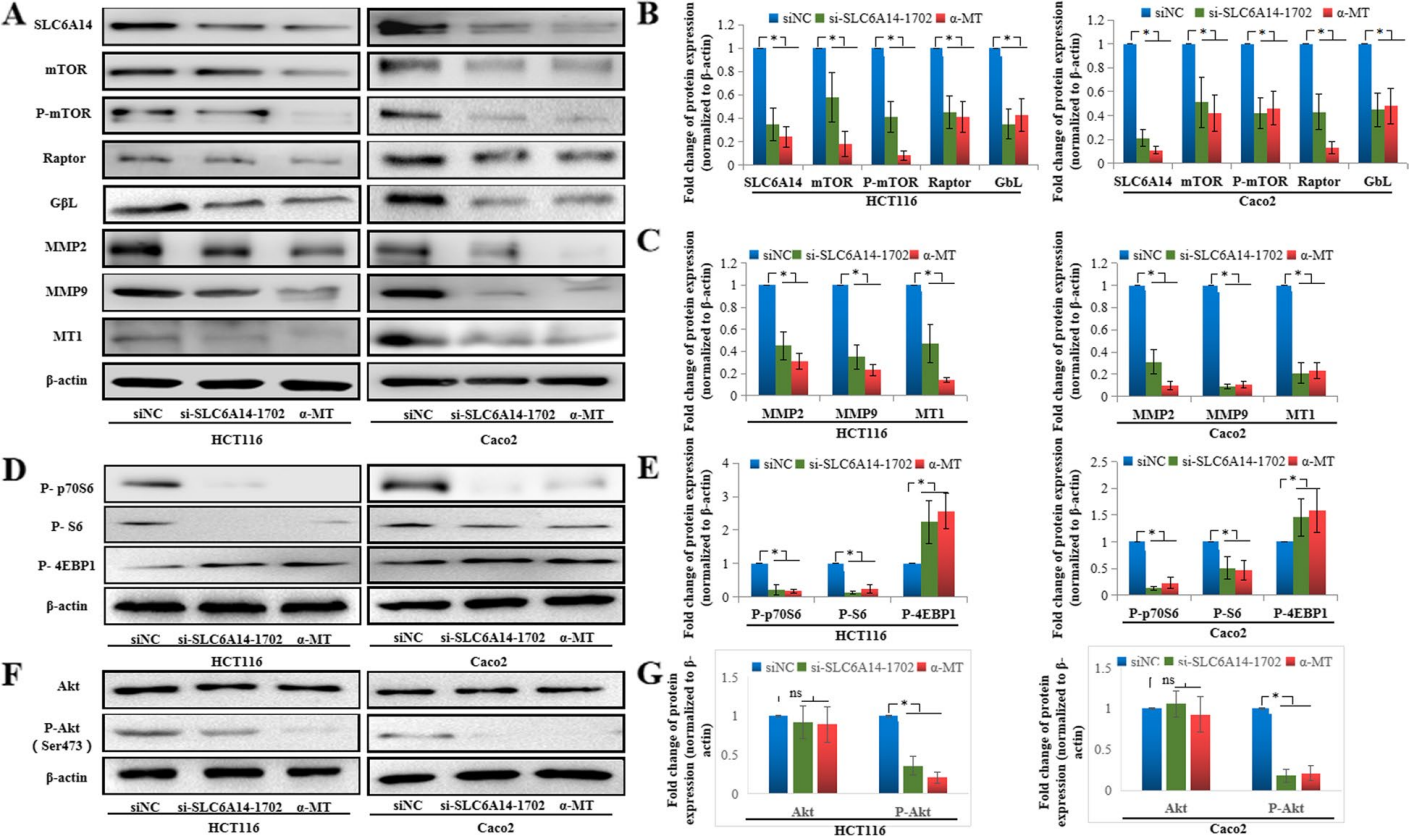
Effects of SLC6A14 on CRC via the activation of mTOR signaling. **A** The protein expression of mTOR signaling and MMPs in the HCT116 or Caco2 cells transfected with SLC6A14-siRNA or blockade of SLC6A14 with α-MT was analyzed by Western blot. **B** The relative protein levels of SLC6A14 and mTOR signaling molecules, and β-actin was used as the internal control. **C** The relative protein levels of MMP2, MMP9, and MT1-MMP, and β-actin was used as the internal control. **D** Western blot analysis was used to determine the downstream effectors of mTOR in HCT116 or Caco2 cells transfected with SLC6A14-siRNA or blockade of SLC6A14 with α-MT. **E** The relative protein levels of P-p70S6, P-S6 and P-4EBP1, and β-actin was used as the internal control. **F** Western blot analysis was used to determine the total-Akt and phosphorylated-Akt (Ser473) in HCT116 or Caco2 cells transfected with SLC6A14-siRNA or blockade of SLC6A14 with α-MT. **G** The relative protein levels of Akt and P-Akt, and β-actin was used as the internal control. The data are expressed as the mean ± SD of three independent experiments. *P < 0.05

To determine the signaling pathway related to the regulation of CRC cell growth mediated by SLC6A14, several Cell Signaling Technology Pathway Antibody Sampler Kits (CST, MA, USA) were used to compare the activities of common cancer-related cell signaling pathways between the SLC6A14 knockdown and control cells, including the p38/MAPK signaling pathway, Notch activated signaling pathway, mTOR signaling pathway, and matrix remodeling. mTOR signaling activities were found to be affected significantly. As shown in Fig. 6A and B, the expression of total mTOR, phosphorylated mTOR (Ser2448), GβL, and Raptor was downregulated in the SLC6A14 knockdown cells or treatment with α-MT compared to the control HCT116 or Caco2 cells. mTOR, GβL, and Raptor constitute an important signaling pathway in the regulation of tumor growth [29, 30]. Furthermore, downstream effectors of mTOR were investigated by western blot. As shown in Fig. 6D and E, phosphorylation levels of p70S6 and S6 were downregulated in the SLC6A14 knockdown cells or treatment with α-MT compared to the control HCT116 or Caco2 cells, but phosphorylation of 4E-BP1 was upregulated. As the mTOR signaling pathway is usually regulated by PI3K/Akt, the protein levels of total Akt and phosphorylated Akt were examined in HCT116 and Caco2 cells. As shown in **Fig.** **6****F and G**, p-Akt (Ser473) was downregulated in the cells treated with either SLC6A14-siRNA or α-MT, while total Akt showed no change.

To further investigate the molecular mechanism underlying the SLC6A14-regulated cell invasion in CRC, we detected matrix remodeling protein expression in HCT116 and Caco2 cells and found that MMP2, MMP9, and MT1-MMP were downregulated by treated with SLC6A14-siRNA or α-MT (Fig. 6A and C).

### Pharmacological blockade of SLC6A14 in vivo inhibits xenograft tumor growth

**Fig. 7:**
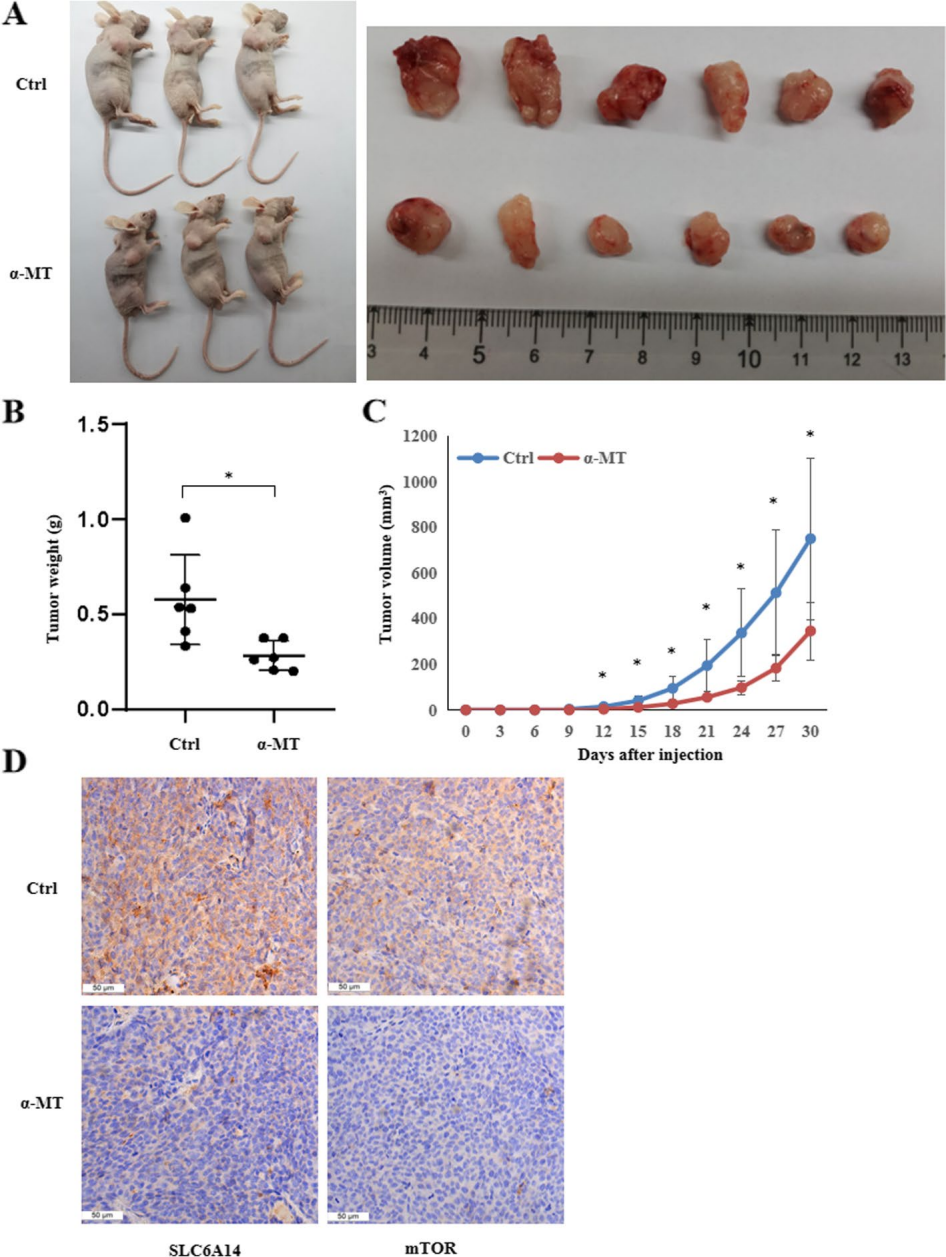
Blockade of SLC6A14 with 2 mg/ml α-MT in vivo inhibits xenograft tumor growth. **A** The nude mice were sacrificed, and the tumor tissues were collected and photographed. **B** The tumor weights were measured. **C** The tumor growth curve was measured. **D** Immunohistochemical staining analysis of SLC6A14 and mTOR protein expression in the tumor tissues from each group, 1 bar = 50 μm. The data are expressed as the mean ± SD. *P < 0.05

We next assessed the role of SLC6A14 in a CRC xenograft mouse model. HCT116 cells were subcutaneously injected into nude mice (*n* = 6), and the effect of α-MT on tumor proliferation was evaluated. As shown in Fig. 7A, B and C, we found that α-MT dramatically inhibited CRC tumor growth in these mice compared to the control group. Immunohistochemical staining confirmed that the tumors from the α-MT-treated mice had much lower SLC6A14 and mTOR protein expression (Fig. 7D). Taken these data together, it suggests that pharmacological blockade of SLC6A14 with α-MT effectively suppresses the in vivo growth of CRC.

## Discussion

As rapid proliferating of cancer cells needs a great number of nutrients to support increased biosynthesis and thus many different nutrient transporters are overexpressed on the cell surface, so metabolic reprogramming is recognized as a new hallmark of cancer cells recently [31–33]. Elevated expression of nutrient transporter proteins in malignant cancers and low expression in normal tissues are ideal targets for selective anticancer therapies [34–36]. SLC6A14 has been shown to be such a cancer-specific amino acid transporter: it is highly expressed in estrogen receptor-positive (ER+) breast cancers and pancreatic cancer, but low in normal tissues [21]. Moreover, SLC6A14 knockout mice have no obvious phenotype and show normal fertility and breast development [22]. α-MT, a small-molecule inhibitor targeting SLC6A14, has been successfully employed to block the function of SLC6A14 in a breast cancer xenograft model. Furthermore, α-MT can inhibit tumor cell proliferation both in vitro and in vivo only in SLC6A14-positive breast cancer lines, but not in SLC6A14-negative breast cancer cell lines [22]. These findings strongly indicate that whether SLC6A14 can be a potential tumor target depends on its expression in this tumor.

Our study demonstrated that the mRNA and protein levels of SLC6A14 were upregulated in CRC tissues, as determined by qPCR and immunohistochemistry arrays, in accordance with the results of the TCGA database. In spite of the expression of some amino acid transporters in our study did not agree with the results of TCGA, such as SLC7A4 and SLC7A6. This may be due to the limited sample size used in our study. A large sample with multi-center prospective cohort studies should be performed to confirm these results. Although the upregulation of SLC6A14 in CRC has been well demonstrated by our data and literature [24, 25], we noticed the low expression level of SLC6A14 in SW480 cells, compared to other CRC cell lines including HT29, HCT116, SW620 and Caco2. This was also reported by Karunakaran S, et al. [37] This is mainly due to the higher differentiation of SW480 cells. We have demonstrated that the expression levels of SLC6A14 were closely related to the tumor cells differentiation: the higher the expression of SLC6A14 was, the poorer the differentiation of the tumor was. In CRC, SLC6A14 shows high expression in poorly-differentiated HCT116 cells and undifferentiated Caco-2 cells, while low in differentiated HT29 and SW480 cells. When Caco2 cells were differentiated to the small intestinal epithelium in vitro, SLC6A14 lost the expression [38]. However, we cultured Caco2 cells in the undifferentiated medium in the current study, showing maintenance of the high level of SLC6A14. Moreover, the relationship between SLC6A14 and clinicopathological variables was further found that the higher the clinical stage was, the higher the expression level of SLC6A14 in CRC was. Subsequently, we provided evidence that knockdown of SLC6A14 expression or treatment with α-MT reduced CRC cell proliferation and migration in vitro through restricting amino acids uptake and inhibited xenograft tumor growth in vivo. It has been well demonstrated that among the 20 amino acids, 18 of them can be transported by SLC6A14 except for L-glutamate and L-aspartate. In addition, nonessential amino acids including L-histidine, L-ornithine, and L-citrulline can be synthesized endogenously by mammalian cells. As a result, these five amino acids in the culture supernatant may not be transported by SLC6A14 or not be required by HCT116 cells. This may explain the reason why histidine, ornithine, citrulline, and aspartic acid in the cell culture supernatant did not show influence by silencing of SLC6A14. Although we observed the increase of glutamate in the culture supernatant following SLC6A14-siRNA treatment or pharmacological blockade of SLC6A14 with α-MT, the mechanism remains to be determined. Taken together, these results highlight that SLC6A14 could be an effective molecular target for the treatment of CRC.

To date, the expression of SLC6A14 is upregulated in estrogen receptor-positive breast cancer, cervical cancer, pancreatic cancer, and colorectal cancer [19–21, 24, 25]. Furthermore, Sikder MOF et al. [27] demonstrated that the tumor-associated up-regulation of SLC6A14 was likely to be driven by Wnt signaling by activating the downstream mediator TCF4/β-catenin. Mao et al. found that SLC6A14 promoted proliferation and metastasis of CRC via enhancing the JAK2/STAT3 pathway [25]. However, little is known about SLC6A14 being involved in downstream signaling pathways in CRC proliferation. In our study, we not only detected the overexpression of SLC6A14 in CRC but also demonstrated that SLC6A14 affected the mTOR signaling pathway in CRC cells. Knockdown of SLC6A14 with siRNA or blockade with α-MT inhibited the activity of mTOR, phosphorylated mTOR (Ser2448), GβL, and Raptor, which constituted rapamycin complex 1 (mTORC1). Moreover, knockdown of SLC6A14 with siRNA or blockade with α-MT decreased phosphorylated p70S6 and S6, but increased phosphorylated 4E-BP1. As we all know, p70S6, S6 and 4E-BP1 are the downstream effectors of mTOR: p70S6 and S6 phosphorylation stimulate protein synthesis; phosphorylated 4E-BP1 inhibits protein synthesis by binding to EIF4E. And mTOR is usually regulated by PI3K/Akt signaling, so Akt is the upstream molecule of mTOR. As shown in our study, p-Akt (Ser 473) was downregulated in the cells treated either with SLC6A14-siRNA or α-MT blockade, while total Akt was not changed. Taken these data together, it suggested that SLC6A14 regulated the activity of Akt-mTOR signaling molecules, which is a key pathway in the control of tumor cell proliferation and mobility [39–41]. This observation is in accordance with the fact that SLC6A14 is sufficient for the active import of almost all of the amino acids from the extracellular medium, among which L-glutamine and L-leucine are key activators of the mTORC1 signaling pathway [42, 43]. It is also reported that SLC38A9, a key component of a lysosomal membrane complex, interacts with the Rag GTPases and Ragulator in an amino acid-sensitive fashion, and activates mTORC1 by amino acids, particularly arginine [44]. Thus, through SLC38A9, arginine serves as a lysosomal messenger that couples mTORC1 activation to the release from lysosomes of the essential amino acids needed to drive cell growth [45].

By application of 482 tissue samples of CRC and three additional CRC cell lines HCT116, Caco2 and SW480, our current study not only further confirmed the findings by Mao et al. and Sikder et al. but also demonstrated a novel regulatory mechanism through which Akt-mTOR signaling mediated the function of SLC6A14 in CRC. In combination with the findings by our study and literature, the molecular mechanisms for SLC6A14 in regulating CRC through Wnt signaling, Akt-mTOR signaling, and JAK2/STAT3 signaling were determined.

In addition, LC-MS/MS analysis was applied in our study to determine concentrations of 20 amino acids in the supernatant of HCT116 cells with or without expression of SLC6A14. As a result, we are the first to find that 16 of the 20 amino acids were increased in the supernate of HCT116 cells after silencing of SLC6A14 with siRNA or blockade with α-MT, demonstrating an important role of SLC6A14 in the regulation of amino acid uptake in CRC cells, which are essential for cancer cell proliferation.

Pharmacological blockade of these nutrient transporters might offer a novel strategy in cancer therapy: “starve the tumor cells to death” [46], so a small-molecule inhibitor with minimal toxicity targeting SLC6A14 is worth seeking. α-MT was identified as a blocker for SLC6A14, which caused 50% blockage of SLC6A14 transport function when the concentrations of all these 18 amino acids were up to ∼250 μM in vivo conditions. α-MT can suppress colony-forming ability and cell cycle arrest in SLC6A14-positive tumor cells, whereas SLC6A14-negative cell lines are not affected [36]. α-MT has been successfully applied to SLC6A14-positive breast cancer xenograft models and significantly suppresses tumor growth [22]. Our results showed that α-MT inhibited CRC cell lines (HCT116 and Caco2) proliferation, promoted cell apoptosis, reduced migration in vitro as expected, and suppressed tumor growth in a nude mouse CRC model. However, the effect of α-MT on SW480, which was low-expressed SLC6A14, did not show any significant change of cell proliferation and cell apoptosis, compared to controls when treated with either SLC6A14-siRNA or α-MT blockade. These results show that α-MT may lead to the deprivation of amino acids in cells dependent on transporters. However, whether α-MT can be a candidate for novel approaches to CRC therapy requires further study on the role of SLC6A14 in a variety of cancers. Moreover, although the single use of α-MT is encouraging, it is more important to assess whether the combination of drugs will lead to greater inhibition and suppress the development of drug resistance.

## Conclusions

In conclusion, our experiments indicated that SLC6A14 was upregulated and could activate the Akt-mTOR signaling pathway, thereby promoting the tumor progression of CRC. Blockade of SLC6A14 with α-MT reduced CRC cell growth in vitro and in vivo. These findings highlight the clinical and therapeutic utility of SLC6A14 to target the constitutive anabolism of CRC for treatment.

## Supplementary Information

**Additional file 1.**

## Data Availability

The datasets generated during the current study are not publicly available but de-identified and anonymized information is potentially available on reasonable request from the corresponding author (Ying Lu).

## Abbreviations

α-MT: α-methyltryptophan
COAD: Colon adenocarcinoma
CRC: Colorectal cancer
CST: Cell signaling technology
DAB: Diaminobenzidine
FBS: Fetal calf serum
GβL: G protein beta subunit like protein
H-score: Histochemistry score
IHC: Immunohistochemistry
LC-MS: Liquid chromatograph-mass spectrometer
mTOR: Mammalian target of rapamycin
OD: Optical density
real-time PCR: Real-time polymerase chain reaction
SLC6A14/ATB0,+: Solute carrier family 6 member 14
TCGA: The cancer genome atlas
