# Supplementary material for: Blockade of the amino acid transporter SLC6A14 suppresses tumor growth in colorectal Cancer
Source: BMC Cancer. 2022 Jul 30;22:833. doi: 10.1186/s12885-022-09935-0 (PMC9339205; doi:10.1186/s12885-022-09935-0)
Supplement: Supplementary file 1 — Additional file 1. [file 12885_2022_9935_MOESM1_ESM.pdf]

Supplementary Figure 1

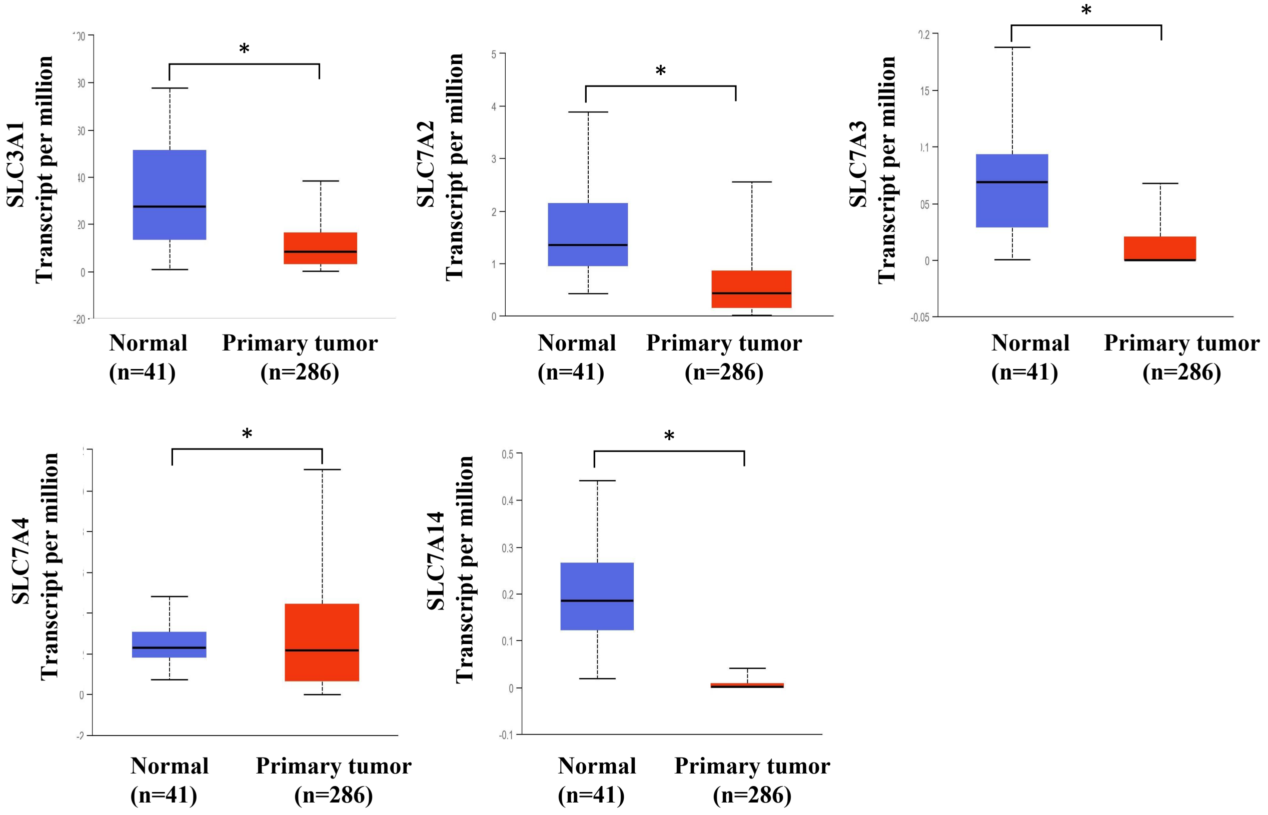

Supplementary Figure 2

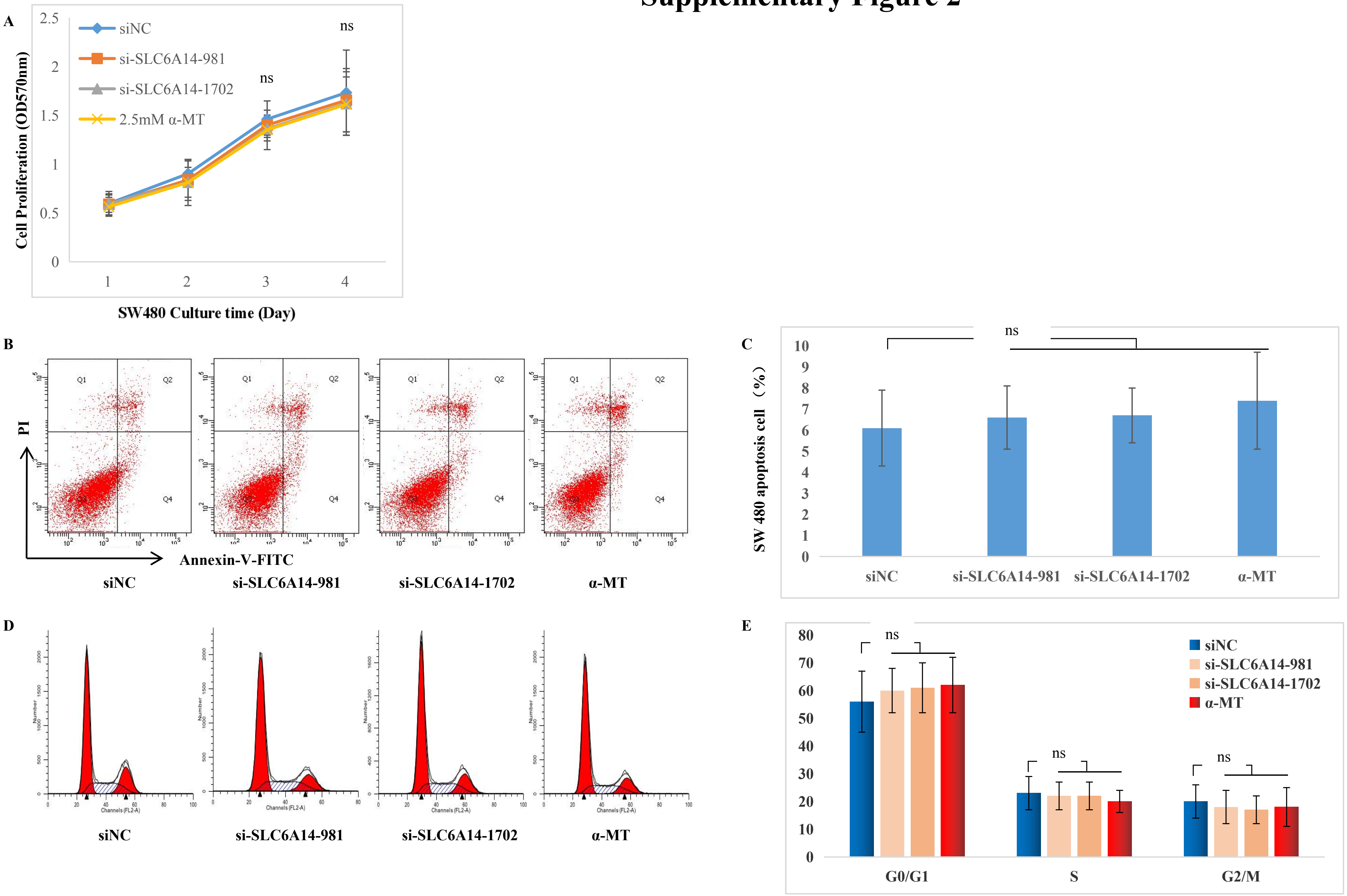

Supplementary Figure 3

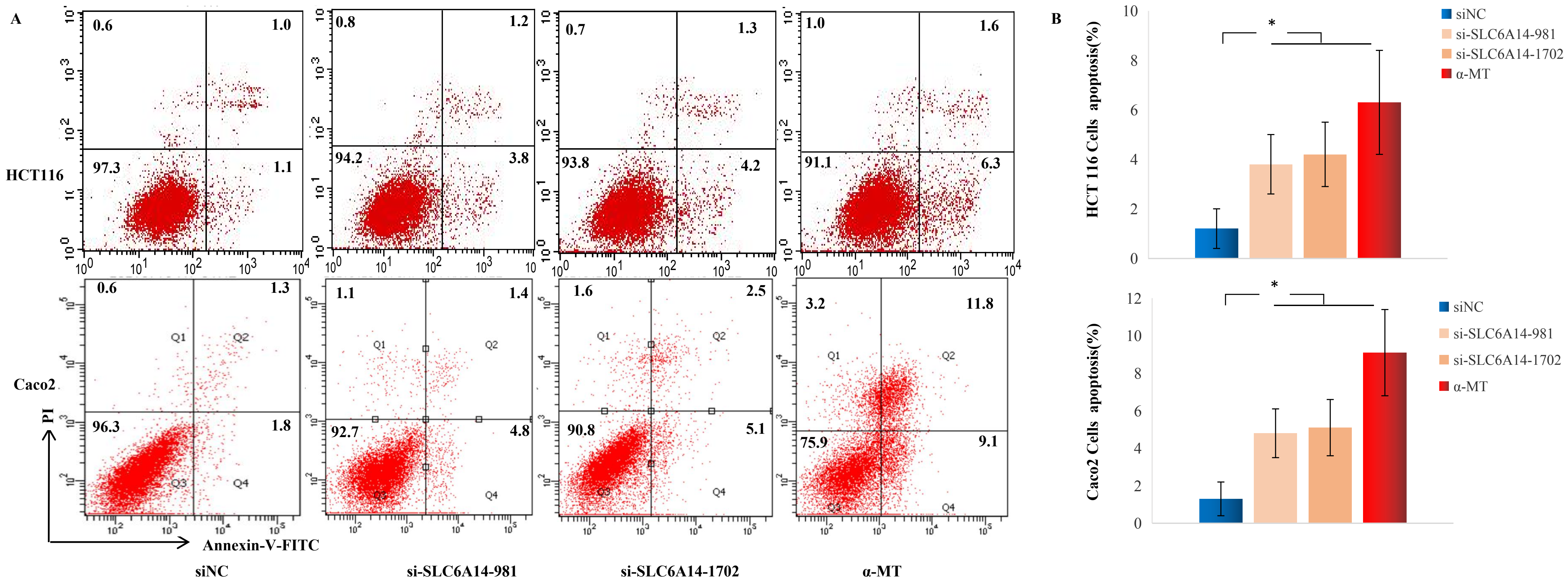

Supplementary Figure 4

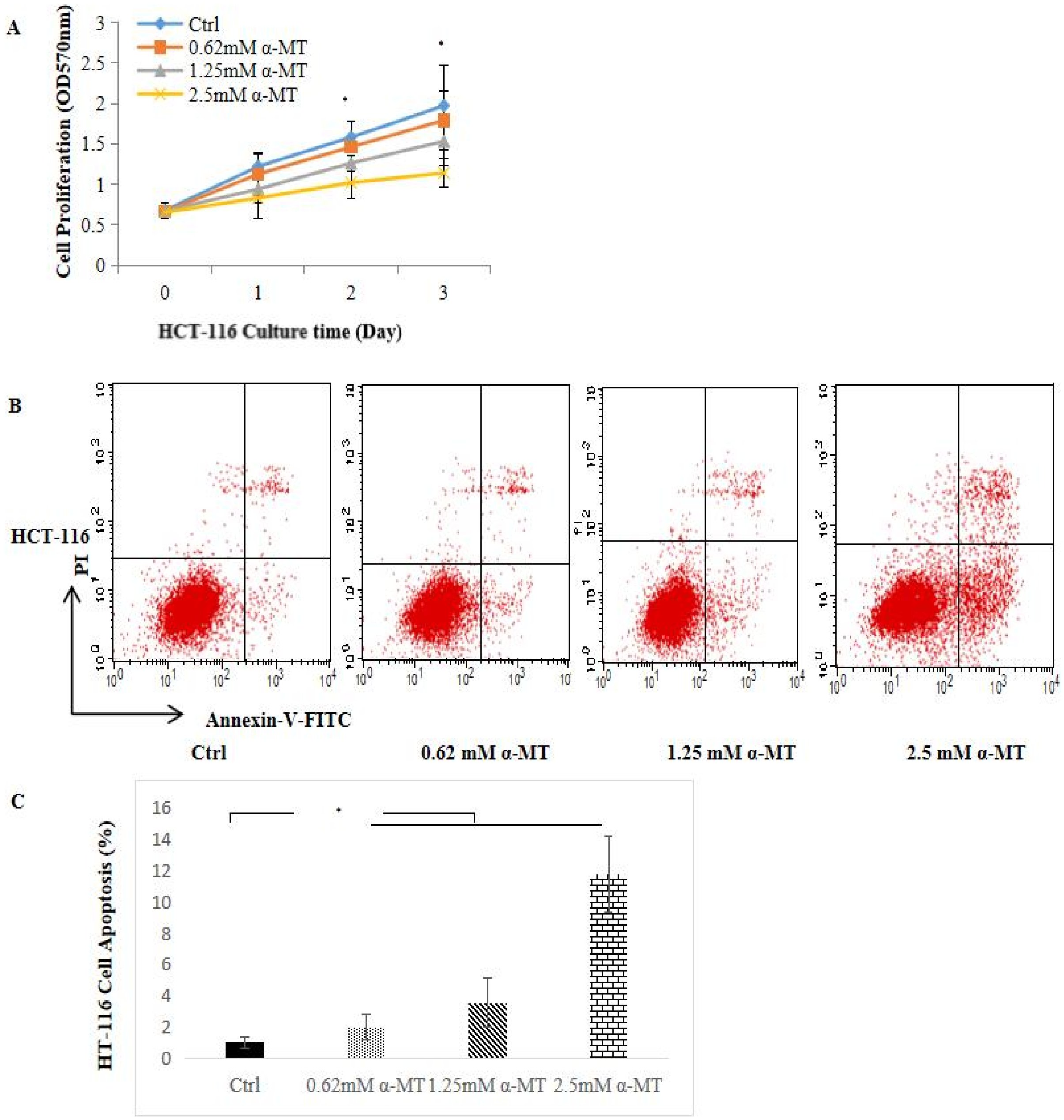

Supplementary Figure 5

Ctrl

α-MT

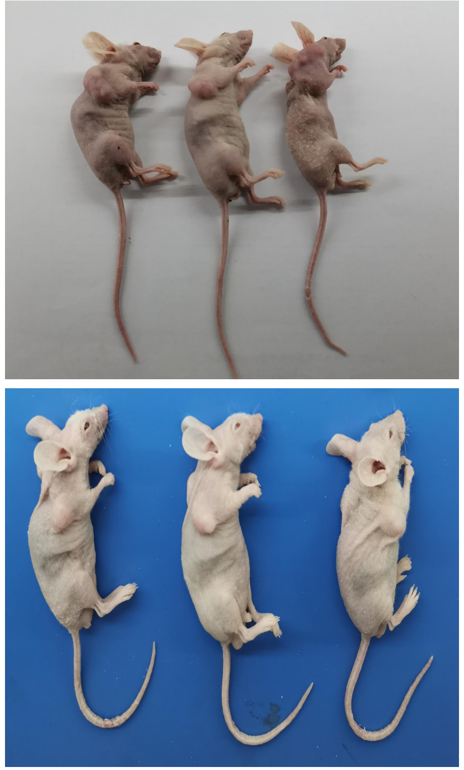

SUPPLEMENTARY FIGURE LEGENDS

Supplementary Fig. 1 The publicly available TCGA database was used to analyze the mRNA expression levels of SLC3A1, SLC7A2, SLC7A3, SLC7A4 and SLC7A14 in colorectal cancer. **P*<0.05.

Supplementary Fig. 2 The effects of knockdown or blockade of SLC6A14 in SW480 cells. a The growth curve of the SW480 cells that were transfected with SLC6A14-siRNA or blockade of SLC6A14 with α-MT, as determined by MTT assay. **b** The cell apoptosis of the SW480 cells transfected with SLC6A14-siRNA or treated with α-MT cultured in FBS-free medium was stained with Annexin-V/PI and examined by flow cytometry. **c** The quantification of apoptotic cells identified as PI-negative and Annexin-V-positive staining. **d** Cell cycle analysis G_1/S transition of the SW480 cells transfected with SLC6A14-siRNA or treated with α-MT by flow cytometry. **e** Quantification of cell cycle analysis. The data are expressed as the mean±SD of three independent experiments.

Supplementary Fig. 3 Knockdown or blockade of SLC6A14 promotes apoptosis in the medium with 10% FBS. a The cell apoptosis of the HCT116 or Caco2 cells transfected with SLC6A14-siRNA or treated with α-MT cultured in 10% FBS medium was stained with Annexin-V/PI and examined by flow cytometry. **b** The quantification of apoptotic cells

identified as PI-negative and Annexin-V-positive staining. The data are expressed as the mean±SD of three independent experiments. *P< 0.05.

Supplementary Fig. 4 The effects of different concentrations of α-MT on cell proliferation and apoptosis. a The growth curve of the HCT116 cells treated with 0mM, 0.62mM, 1.25mM and 2.5mM α-MT, as determined by MTT assay. **b** The cell apoptosis of the HCT116 cells treated with 0mM, 0.62mM, 1.25mM and 2.5mM α-MT was stained with Annexin-V/PI and examined by flow cytometry. **c** The quantification of apoptotic cells identified as PI-negative and Annexin-V-positive staining.

Supplementary Fig. 5 Two pictures of another 6 mice.

WB raw blots
Figure 1

Figure 1C-SLC6A14

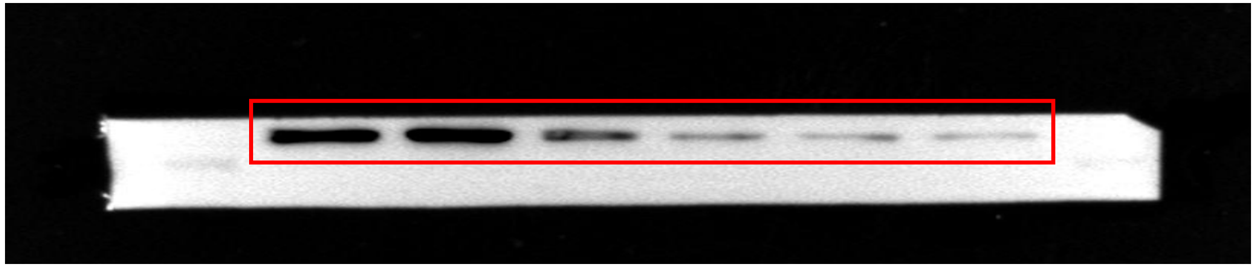

T1 T2 T3 N1 N2 N3

Figure 1E-SLC6A14

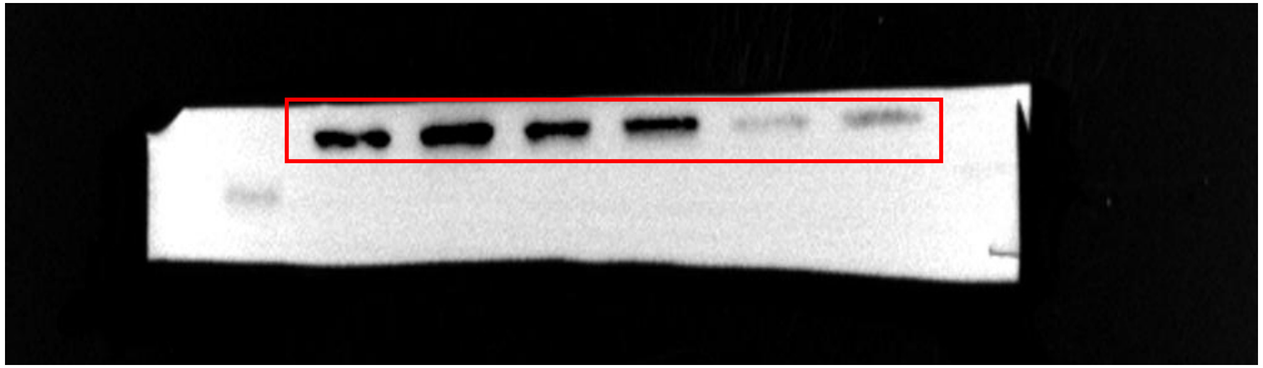

HT29 Caco2 HCT116 SW620 SW480 Huh7

Figure 1E-β-actin

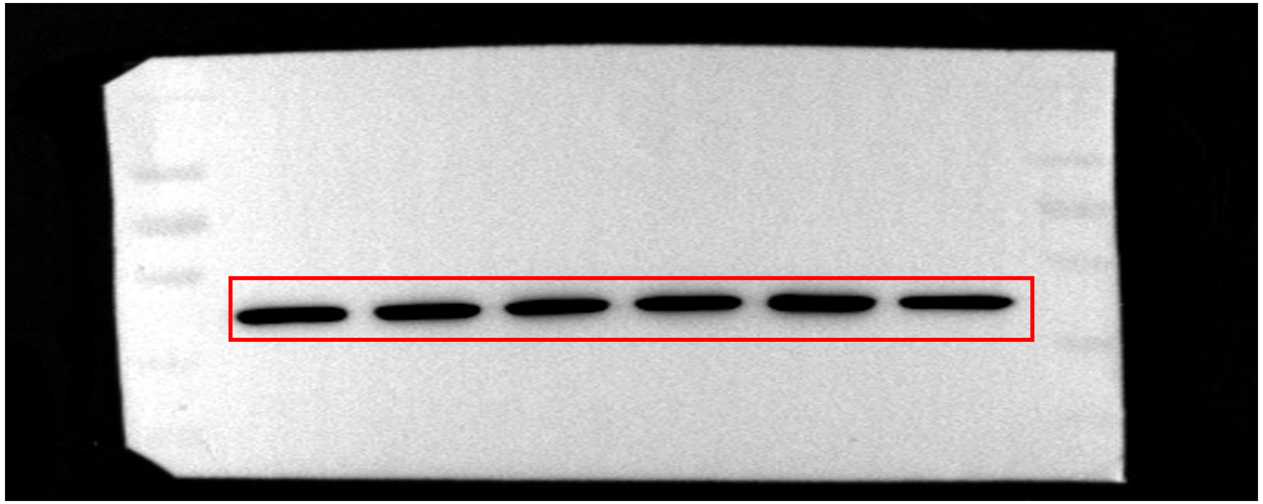

WB raw blots
Figure 3

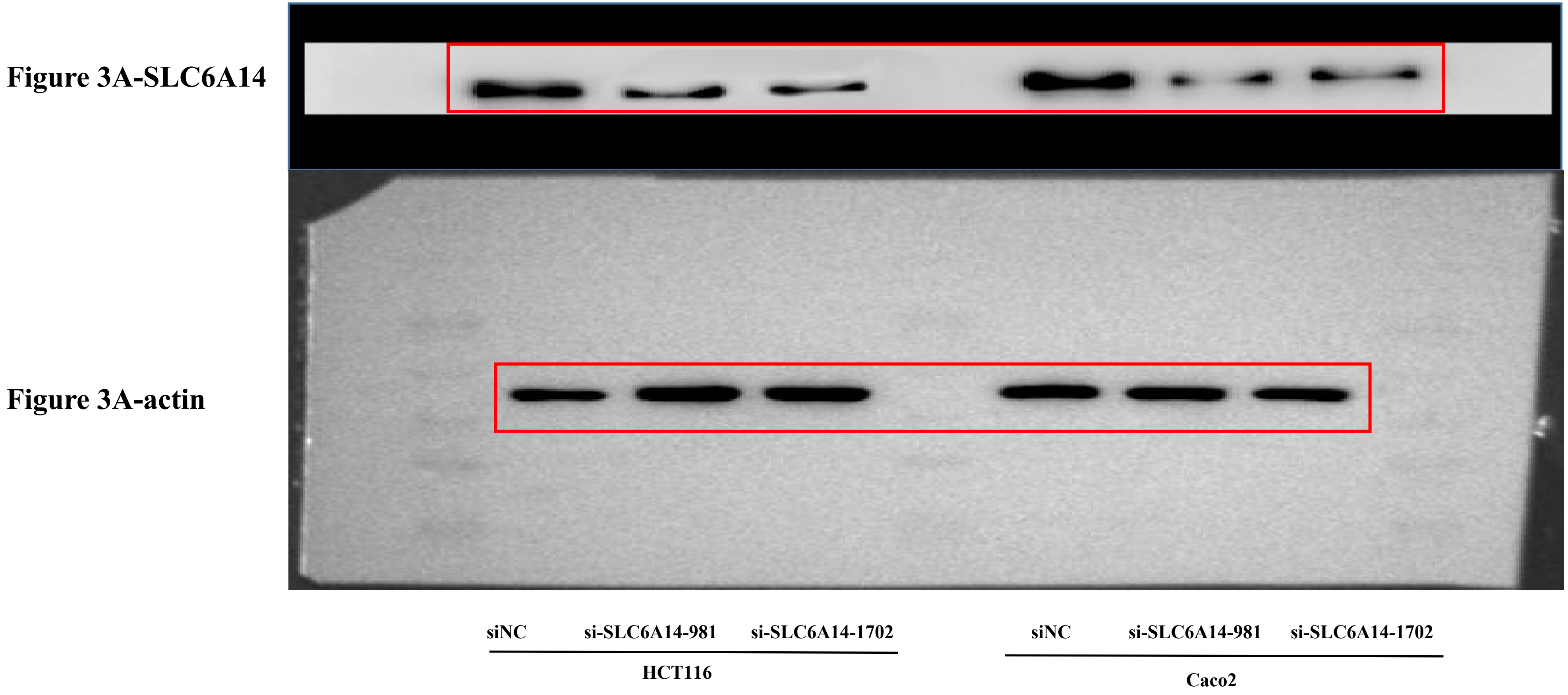

WB raw blots
Figure 6A-HCT116-mTOR

Figure 6 A-mTOR-HCT116

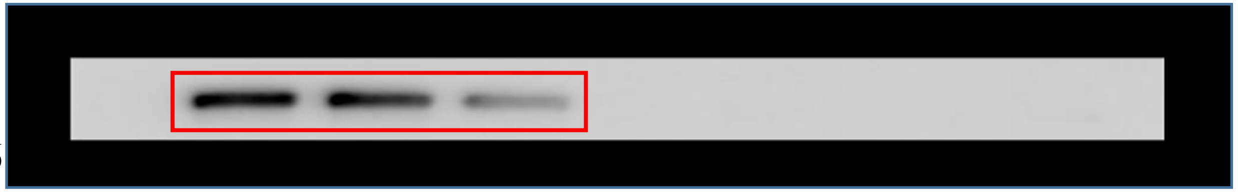

siNC si-SLC6A14-1702 α-MT

Figure 6A-P-mTOR-HCT116

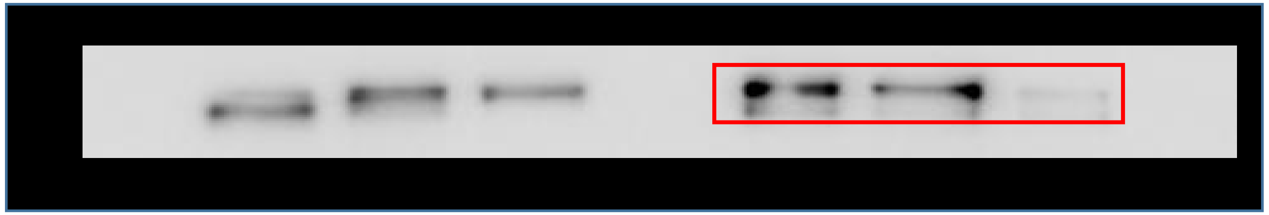

siNC si-SLC6A14-1702 α-MT

Figure 6A-GbL-HCT116

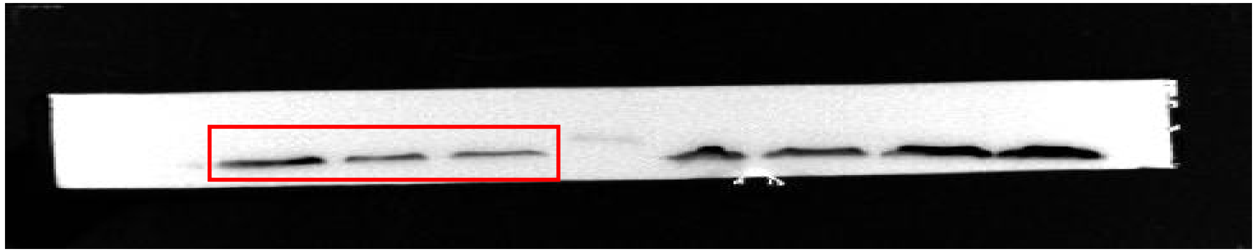

siNC si-SLC6A14-1702 α-MT

Figure 6A-actin-HCT116

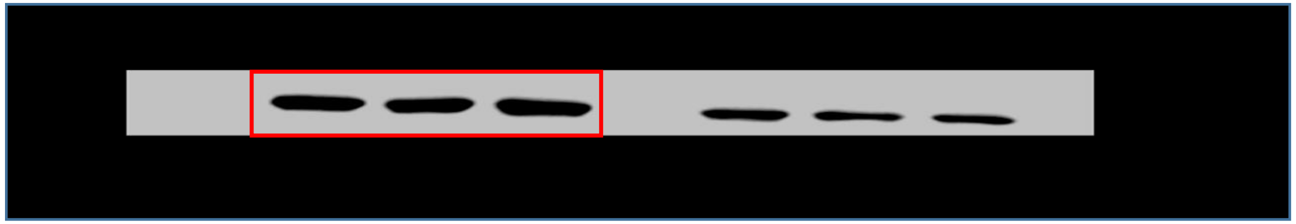

siNC si-SLC6A14-1702 α-MT

Figure 6A-Raptor-HCT116

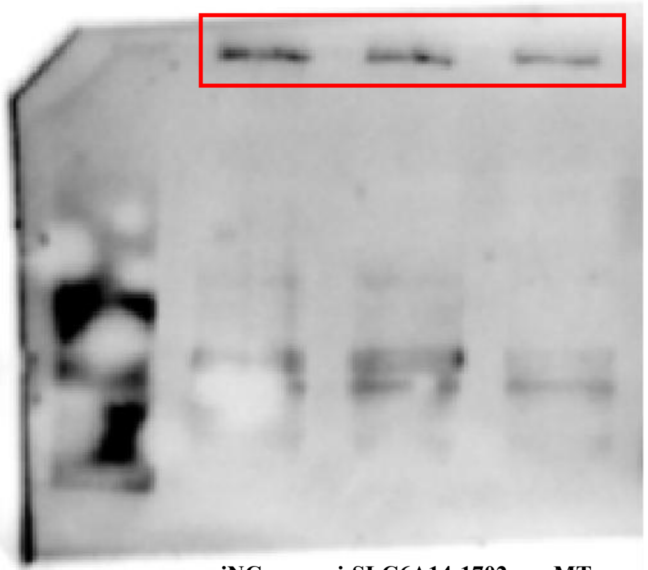

siNC si-SLC6A14-1702 α-MT

WB raw blots
Figure 6A-Caco2-mTOR

Figure 6A-actin-Caco2

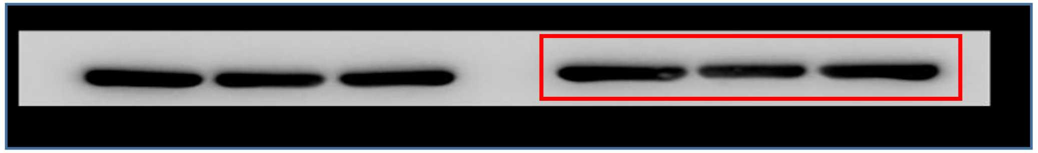

siNC si-SLC6A14-1702 α-MT

Figure 6A-P-mTOR-Caco2

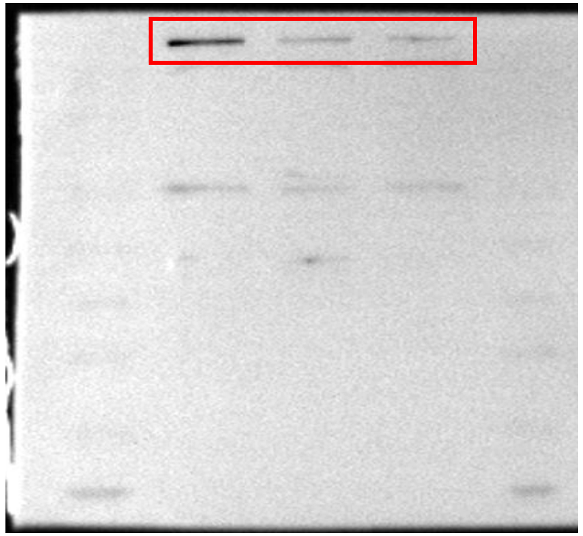

siNC si-SLC6A14-1702 α-MT

Figure 6A-GbL-Caco2

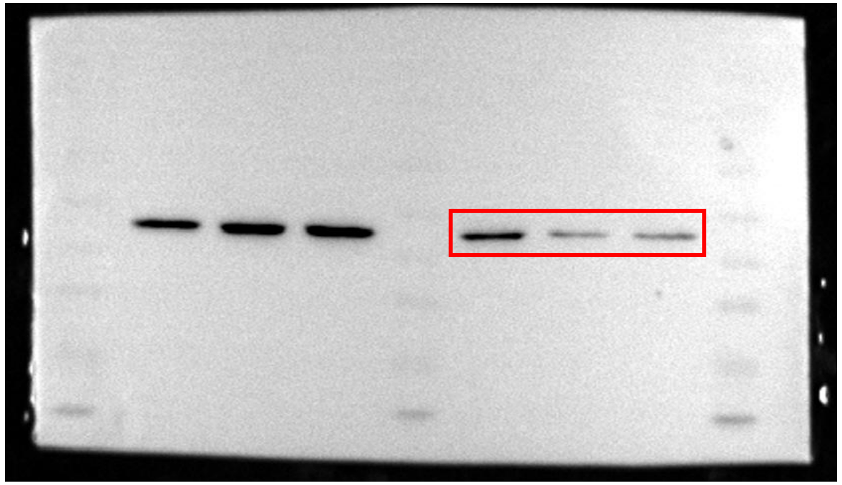

siNC si-SLC6A14-1702 α-MT

Figure 6A-mTOR-Caco2

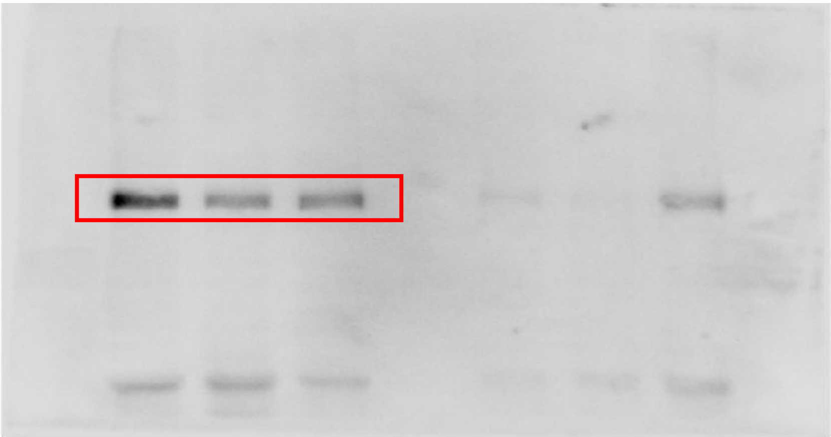

siNC si-SLC6A14-1702 α-MT

Figure 6A-P-Raptor-Caco2

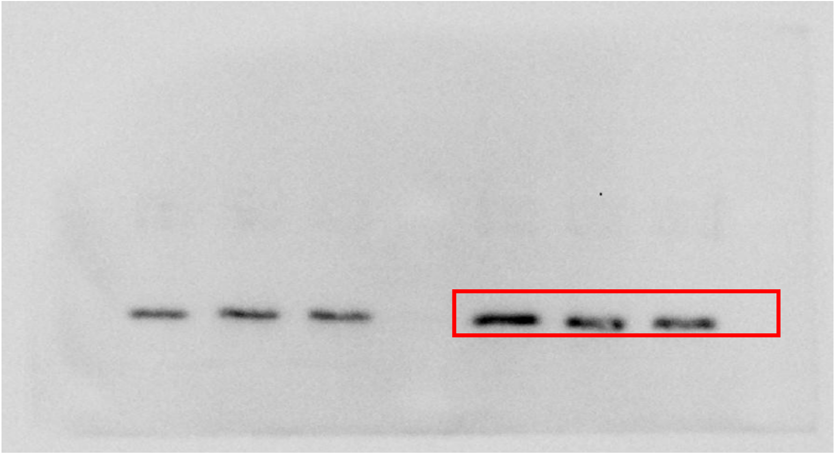

siNC si-SLC6A14-1702 α-MT

WB raw blots
Figure 6A-HCT116-MMPs

Figure 6A-MMP2-HCT116

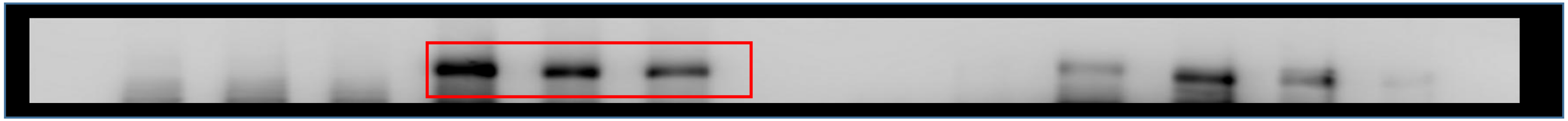

siNC si-SLC6A14-1702 α-MT

Figure 6A-MMP9-HCT116

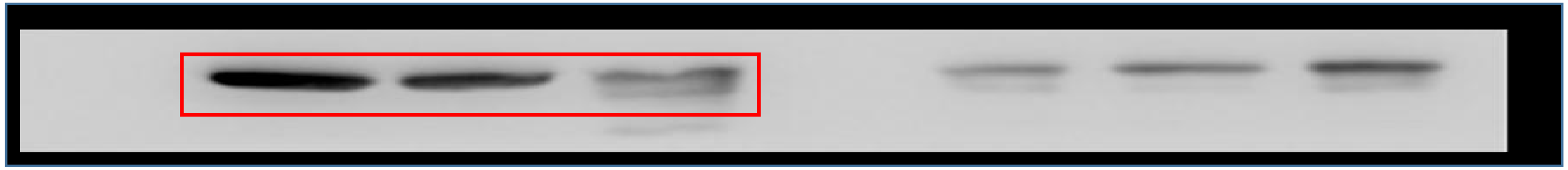

siNC si-SLC6A14-1702 α-MT

Figure 6A-MT1-HCT116

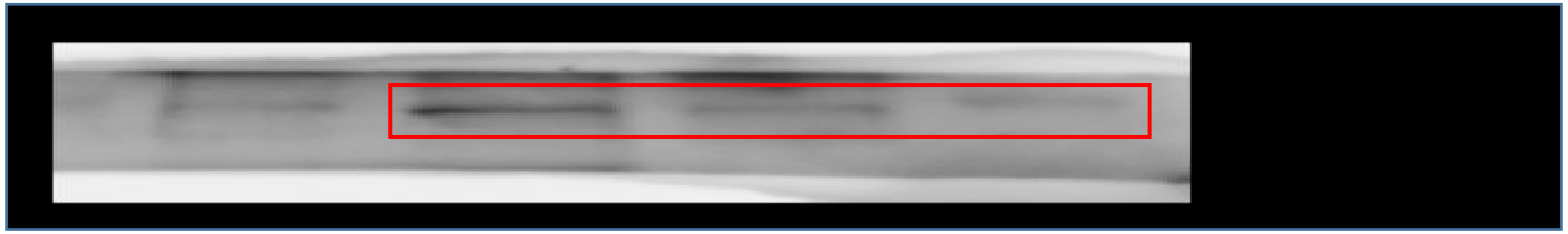

siNC si-SLC6A14-1702 α-MT

Figure 6A-SLC6A14-HCT116

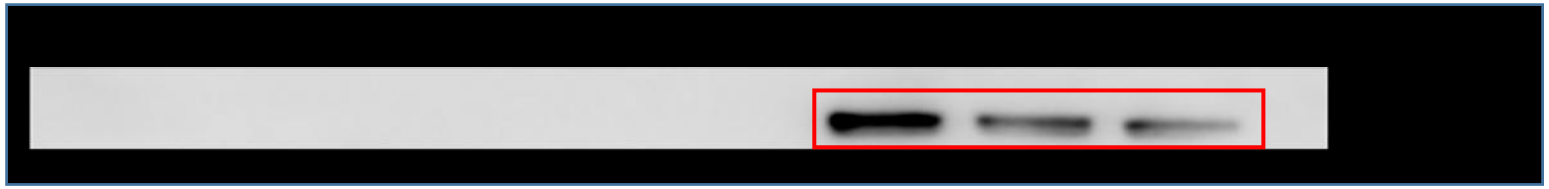

siNC si-SLC6A14-1702 α-MT

WB raw blots
Figure 6A-Caco2-MMPs

Figure 6A-MMP2-Caco2

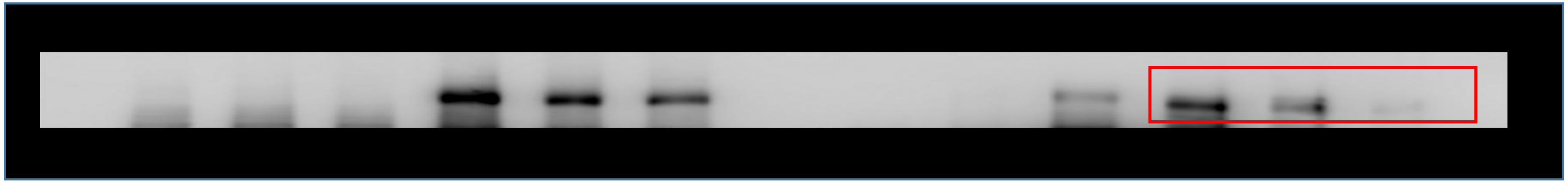

siNC si-SLC6A14-1702 α-MT

Figure 6A-MMP9-Caco2

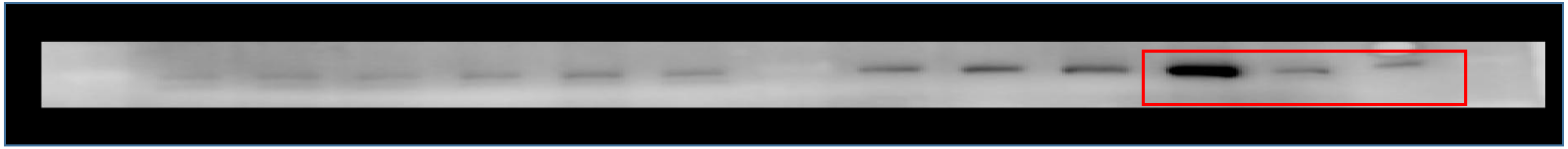

siNC si-SLC6A14-1702 α-MT

Figure 6A-MT1-Caco2

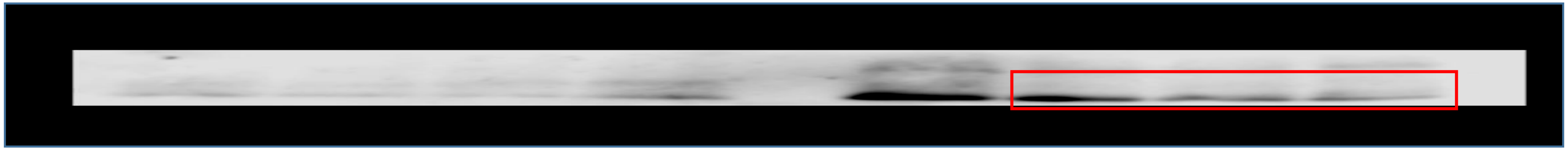

siNC si-SLC6A14-1702 α-MT

Figure 6A-SLC6A14-Caco2

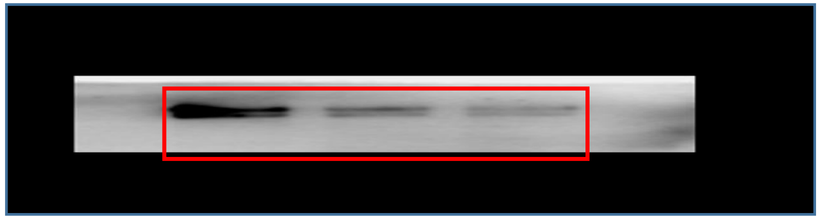

siNC si-SLC6A14-1702 α-MT

WB raw blots
Figure 6D-HCT116

Figure 6D-P-p70S6-HCT116

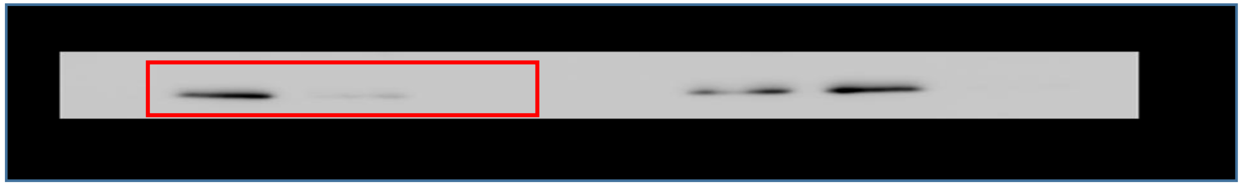

siNC si-SLC6A14-1702 α-MT

Figure 6D-P-S6-HCT116

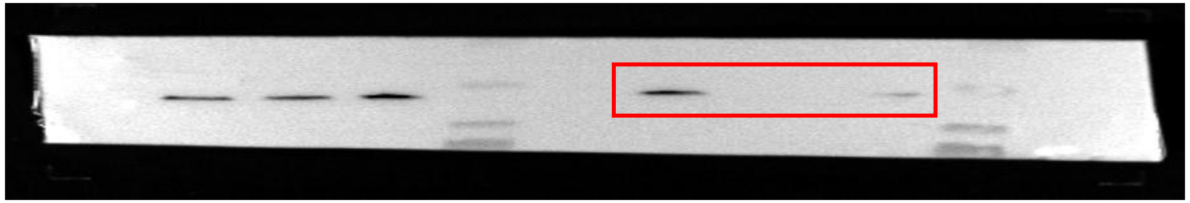

siNC si-SLC6A14-1702 α-MT

Figure 6A-P-4EBP1-HCT116

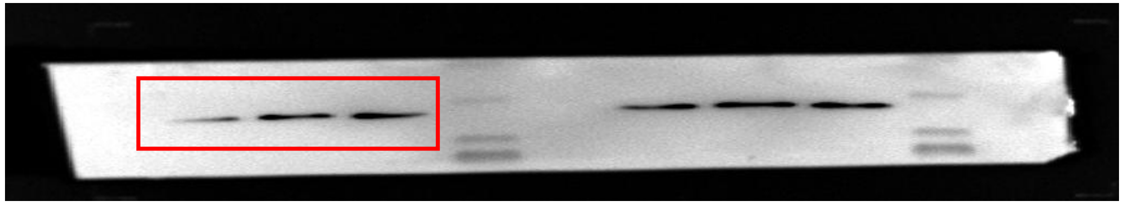

siNC si-SLC6A14-1702 α-MT

Figure 6D-actin-HCT116

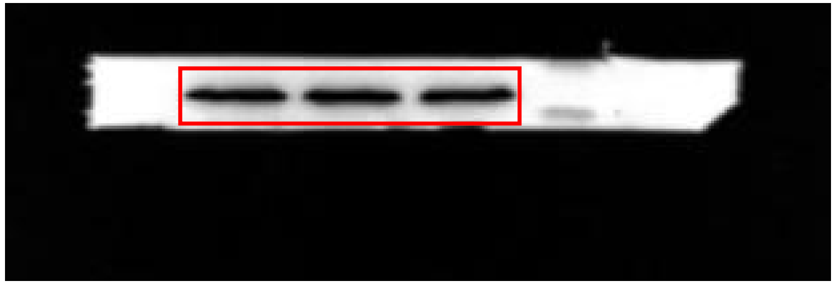

siNC si-SLC6A14-1702 α-MT

WB raw blots
Figure 6D-Caco2

Figure 6D-P-p70S6-Caco2

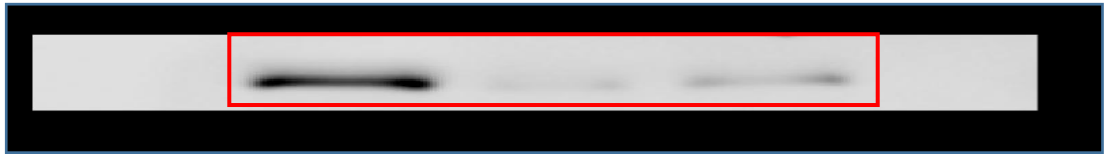

siNC si-SLC6A14-1702 α-MT

Figure 6D-P-S6-Caco2

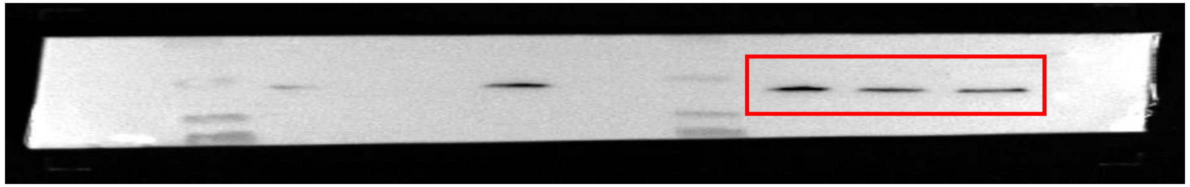

siNC si-SLC6A14-1702 α-MT

Figure 6D-P-4EBP1-Caco2

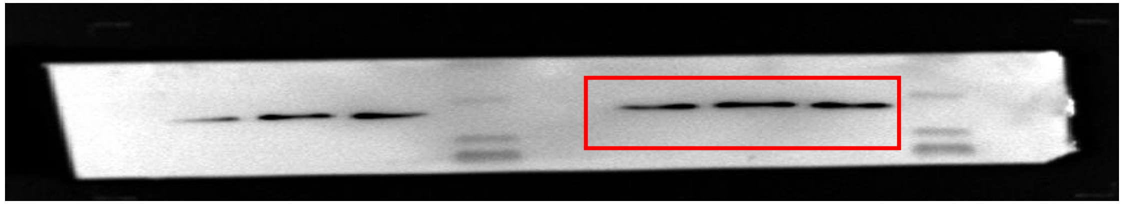

siNC si-SLC6A14-1702 α-MT

Figure 6D-actin-Caco2

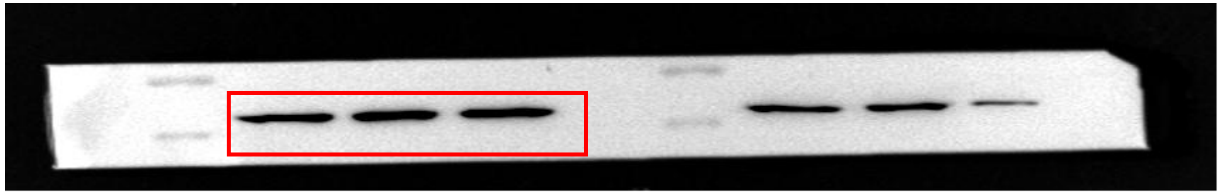

siNC si-SLC6A14-1702 α-MT

WB raw blots
Figure 6F-HCT116

Figure 6F-Akt-HCT116

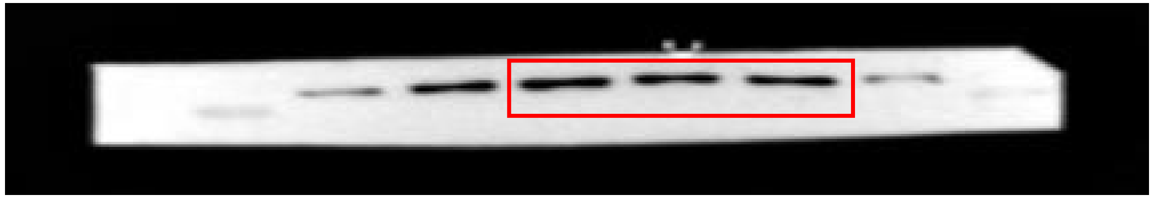

siNC si-SLC6A14-1702 α-MT

Figure 6F-P-Akt-HCT116

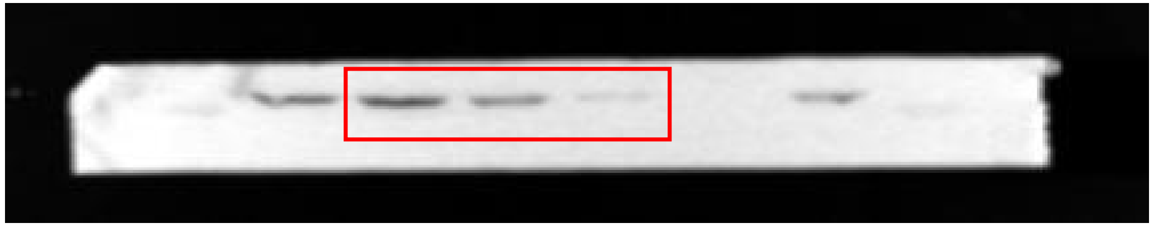

siNC si-SLC6A14-1702 α-MT

Figure 6F-actin-HCT116

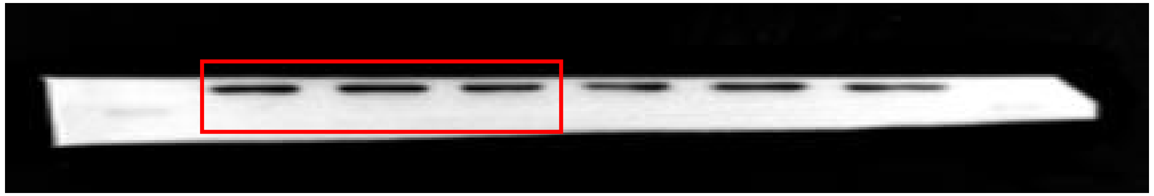

siNC si-SLC6A14-1702 α-MT

WB raw blots
Figure 6F-Caco2

Figure 6F-Akt-Caco2

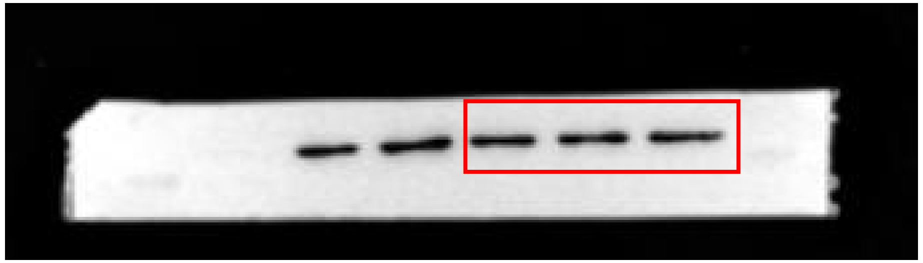

siNC si-SLC6A14-1702 α-MT

Figure 6F-P-Akt-Caco2

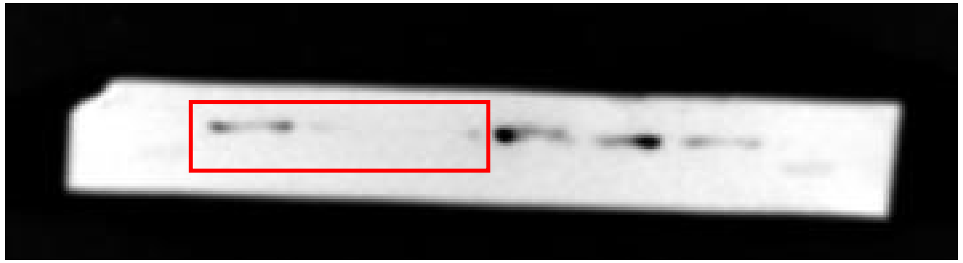

siNC si-SLC6A14-1702 α-MT

Figure 6F-actin-Caco2

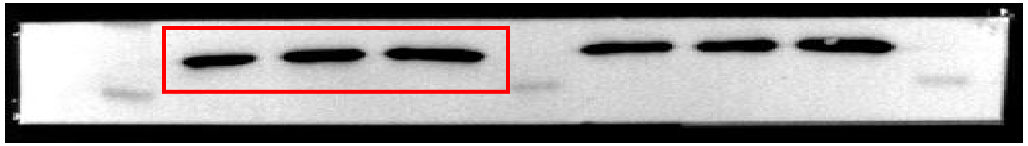

siNC si-SLC6A14-1702 α-MT

WB raw blots
multiple exposure images

exposure time = 30s

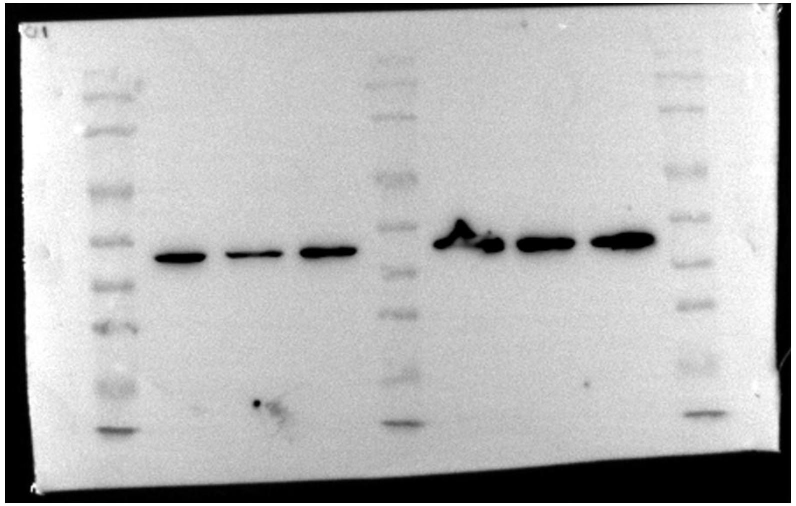

exposure time = 60s

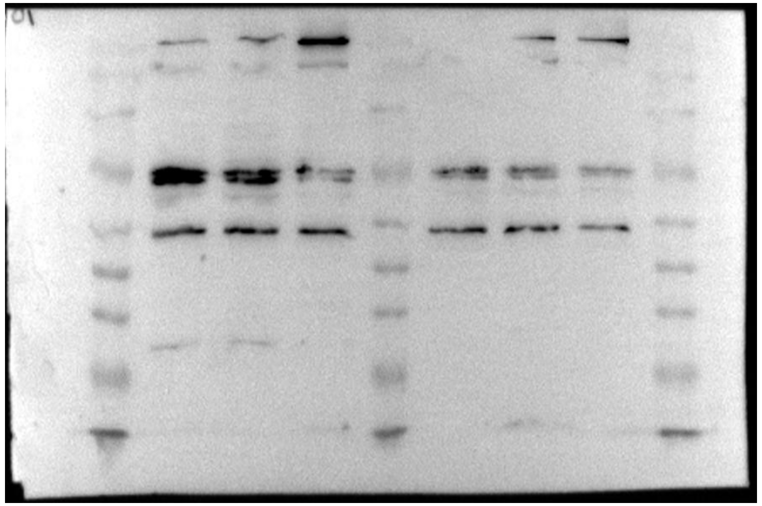

exposure time = 60s

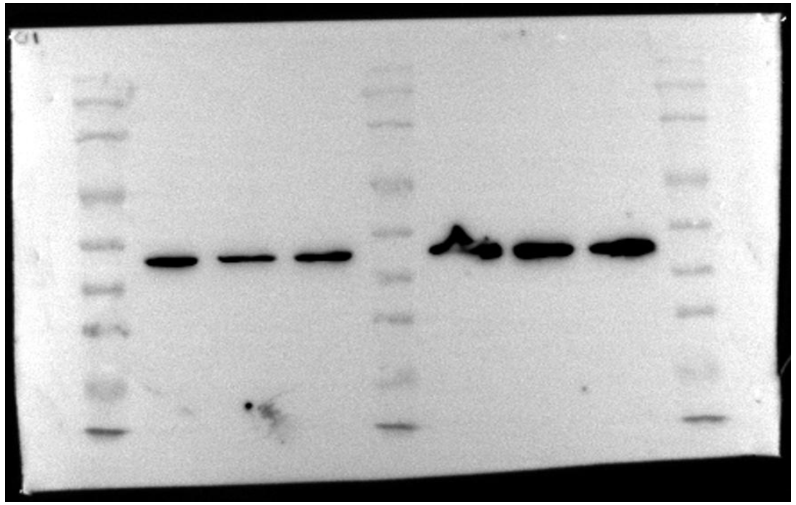

exposure time = 120s

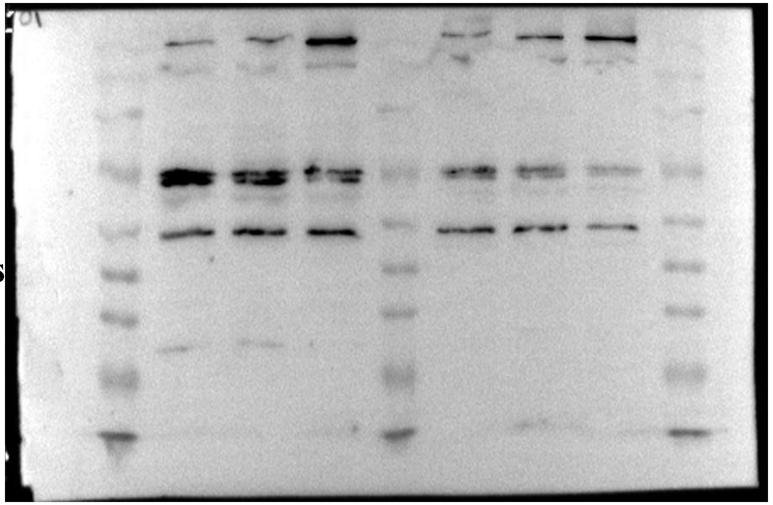

β-actin

SLC6A14
